# Raspberry-Like Plasmonic Nanoaggregates with Programmable Hierarchical Structures for Reproducible SERS Detection of Wastewater Pollutants and Biomarkers

**DOI:** 10.1021/acs.analchem.4c03533

**Published:** 2024-10-24

**Authors:** Huimin Xie, Shuyu Zhu, Ping Wen, Deyue Zhou, Yidan Yin, Yang Lan, Tung-Chun Lee, Yuewen Zhang, Qiaosheng Pu

**Affiliations:** † College of Chemistry and Chemical Engineering, Lanzhou University, Lanzhou 730000, China; ‡ Institute for Materials Discovery, University College London, London WC1H 0AJ, U.K.; § Department of Chemical Engineering, University College London, London WC1E 7JE, U.K.

## Abstract

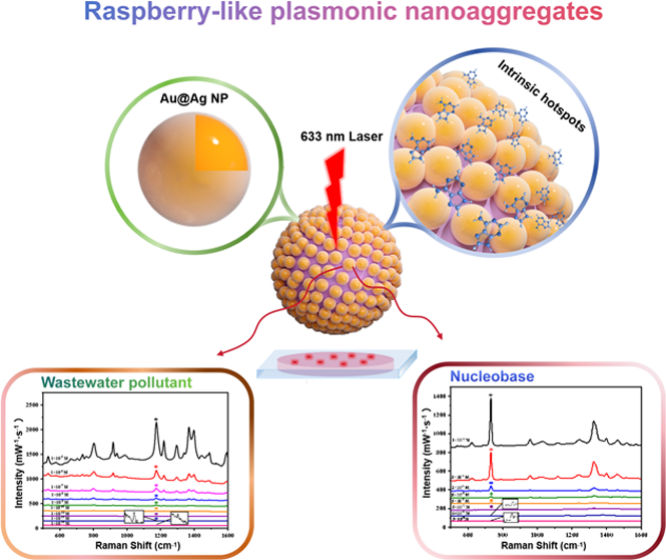

Conventional solid-based SERS substrates often face challenges with inconsistent sample distribution, while liquid-based SERS substrates are prone to aggregation and precipitation, resulting in irreproducible signals in both cases. In this study, we tackled this dilemma by designing and synthesizing raspberry-like plasmonic nanoaggregates that exhibit a high density of hotspots and are colloidally stable at the same time. In particular, the nanoaggregates consist of a core made of functionalized polystyrene (PS) microspheres, which act as a template for rapid self-assembly of Au@Ag core–shell nanoparticles to form raspberry-like hierarchical nanoaggregates within 5 min of mixing. The optimized nanoaggregates can be used as reproducible and stable SERS substrates for a range of wastewater pollutants (e.g., rhodamine 6G (R6G) and malachite green (MG)) and nucleobases (e.g., adenine and uracil), with the detection limits as low as 1 × 10^–10^, 1 × 10^–16^, 3 × 10^–8^, and 3 × 10^–7^ M, respectively. Additionally, the trace detection of adenine in clinical urine samples has been successfully demonstrated. Our modular assembly approach opens up new possibilities in SERS substrate design and advanced trace-chemical detection technologies.

Surface-enhanced Raman spectroscopy (SERS) is increasingly recognized as a versatile analytical technique due to its unique advantages, such as ultrasensitivity, resistance to photobleaching, chemical specificity, narrow Raman spectral bands, and nondestructive analysis.^1,2^ It has been widely used in various fields, including environmental monitoring,^3^ food safety,^4^ and biochemical sensing.^5^

In SERS, the enhancement of Raman signals can be achieved by two mechanisms: chemical enhancement and electromagnetic enhancement.^6^ Compared to chemical enhancement, SERS is strongly influenced by the presence of strong electromagnetic fields near metal nanoparticles.^7^ This has spurred extensive research into the preparation of sensitive SERS substrates.^8^ For precise molecule detection, SERS substrates must exhibit numerous hotspots^9^ that amplify local electromagnetic field strength and provide a significant surface area for adsorbing more molecules, thus enhancing Raman signal strength. However, only a few metals demonstrate a potent SERS effect, and the aggregation of metal nanoparticles often leads to signal instability, posing challenges for quantitative detection. Additionally, solid-based Raman substrates frequently exhibit uneven sample distribution,^10^ underscoring the critical need to develop SERS substrates with improved sensitivity, stability, and quantitative detection capabilities.

To enhance the sensitivity and reproducibility of SERS measurements, a multitude of gold and silver nanomaterials (Au NPs and Ag NPs) with distinct morphologies (e.g., nanorods,^11^ nanotriangles,^12^ nanocone,^13^ and nanocubes^14^) have been successfully synthesized. Au NPs, in particular, are simpler to synthesize, exhibit improved biocompatibility, and offer long-term stability, albeit with a lower SERS enhancement compared to Ag NPs.^15^ Bimetallic Au/Ag NPs capitalize on the strengths of both materials, resulting in stronger SERS signals than pure Au or Ag metals.^1^ Among various bimetallic structures,^16^ core–shell bimetallic nanoparticles typically demonstrate superior physical and chemical properties.^17,18^ For instance, Au@Ag NPs consist of a Au core enveloped by a Ag shell, with the localized surface plasmon resonance (LSPR) of this core–shell structure being highly sensitive to the core size and shell thickness. This addresses issues related to the instability of Ag NPs and the less intense enhancement effects of Au NPs.^19^ By adjusting the thickness of the outer layer, the plasmonic coupling between the bimetals can produce a strong SERS enhancement effect.^20^

Reproducing spectra with SERS poses a persistent challenge due to various factors, including substrate surface morphology and the arrangement of target molecules adsorbed onto the substrate. The raspberry-like nanostructures are composed of closely packed Au NPs assembled on a polymer core. Due to the abundant hotspots between adjacent Au NPs, bright and consistent Raman signals were observed from raspberry-like nanostructures. These findings provide crucial insights into the design of SERS substrates with bright and reproducible signals.^21^ Various synthetic strategies have been proposed to design robust raspberry-like nanostructures, including seed-mediated growth and layer-by-layer (LBL) deposition.^22^ Rong et al.^23^ employed a combination of two surfactants to synthesize Au NPs with a raspberry-like morphology, exhibiting higher SERS activity than smooth-surfaced spherical Au NPs. Yu et al.^24^ synthesized raspberry-like Au NPs using seed-mediated growth, which produced strong and reproducible SERS signals, but this method required subsequent metal-shell growth and additional presynthesis steps. The LBL method is more likely to produce nanoparticle shells by stacking metal nanoparticles on the core surface through electrostatic interactions.^25^ Wang et al.^26^ synthesized raspberry-like PDA@Ag NPs using polydopamine (PDA) spheres as templates, chelating agents, and binding/reducing agents. Unlike seed-mediated growth, PDA microspheres played multiple roles, simplifying the synthesis steps and improving the efficiency. However, the bonding process between metal particles and PDA microspheres still requires specific time and temperature conditions. Consequently, it is beneficial to devise a faster and simpler synthesis method to facilitate the reproducible synthesis of raspberry-like nanoaggregates and obtain consistent SERS signals.

In this study, we devised a straightforward, yet effective, approach to improve SERS sensitivity and reproducibility. In particular, we demonstrate that highly active and colloidally stable solution-based SERS substrates can be rapidly produced within 5 min via aqueous self-assembly of Au@Ag NPs using positively charged, multicarbonyl-functionalized polystyrene (PS) core–shell microspheres as templates, resulting in novel raspberry-like nanoaggregates through electrostatic interactions (Scheme 1). Notably, our modular approach enables facile optimization of the nanoaggregates’ SERS performance through independently tuning the structural parameters of individual components (e.g., thickness of the Ag shell and surface chemistry of the PS microspheres) followed by combining them in a mix-and-match fashion. The optimized nanoaggregates can be used as reproducible and stable SERS substrates for a range of wastewater pollutants (e.g., R6G and MG, selected for their extensive range of applications and potential environmental impacts) and nucleobases (e.g., adenine and uracil, chosen for their importance in the quantitative detection of nucleobases in bioanalytical chemistry and metabolic engineering), with the detection limits as low as 1 × 10^–10^, 1 × 10^–16^, 3 × 10^–8^, and 3 × 10^–7^ M, respectively. Furthermore, the nanoaggregates can also be used for quantifying adenine in undiluted artificial urine down to micromolar concentrations. Spiked assays in clinical urine samples demonstrated satisfactory recoveries (85.87–114.82%) and relative standard deviation (RSD), highlighting their potential for clinical applications. We envision that our hierarchical assembly approach will inspire new SERS substrate designs and open up new possibilities in advanced trace-chemical detection technologies.

**Scheme 1:**
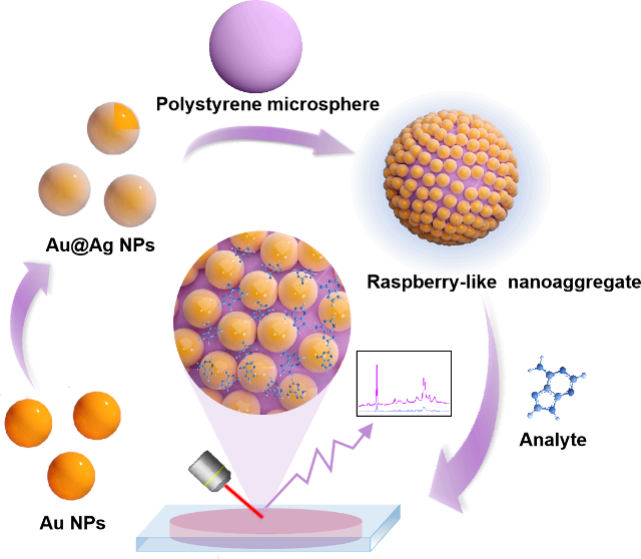
Assembly Process of Au@Ag NPs with PS Microspheres to Form Raspberry-Like Nanoaggregates and Final Structure of the SERS Detection Platform

## Experimental Section

### Synthesis of Au NPs

The synthesis of Au seed involved generating Au NPs with diameters of 30, 40, and 50 nm, following the protocol published by Bastús et al.^27^ 50 mL of 2.2 mM sodium citrate was heated in an oil bath with vigorous stirring. A condenser was utilized to prevent the evaporation of the solvent. After boiling, 335 μL of 25 mM HAuCl_4_ was introduced into a three-necked flask, and the solution was maintained at 90 °C for 30 min. Sequentially, 335 μL of 60 mM sodium citrate and 335 μL of 25 mM HAuCl_4_ were added to the flask (with time delay of approximately 2 min) with stirring at 700 rpm for 30 min until the solution turned ruby red. By the addition of more sodium citrate and HAuCl_4_, larger Au NPs were obtained. The resulting Au NPs colloid was then centrifuged and redispersed to the original volume to remove unreacted reagents from the solution for further synthesis of core–shell Au@Ag NPs.

### Synthesis of Core–Shell Au@Ag NPs

The synthesis of core–shell Au@Ag NPs involved transferring 45 mL of the above Au NPs with diameters of 30, 40, and 50 nm into a three-neck flask. To prevent evaporation, a condenser was employed. The oil bath temperature was regulated at 70 °C while maintaining a stirring speed of 700 rpm. At 30 min intervals, 400 μL of 60 mM sodium citrate and 400 μL of 27 mM AgNO_3_ were added to increase the thickness of the Ag shell (with time delay of approximately 2 min).

### Synthesis of Raspberry-Like Nanoaggregates

Polystyrene microspheres were initially synthesized by emulsion polymerization, and subsequently, five functionalized core–shell microspheres were obtained by adding different ratios of functionalized monomers (AMH, AEMA) for secondary growth on their surface. The raspberry-like nanoaggregates were obtained by mixing 150 μL of Au@Ag NPs solution with 1 μL of each of the five different core–shell microsphere stock solutions in a centrifuge tube. The solution color shift from ruby red to purple within 5 min indicated the self-assembly of Au@Ag NPs with microspheres into raspberry-like nanoaggregates.

### Characterization

Ultraviolet–visible (UV–vis) spectra of the samples were obtained using a microplate reader (Tecan Spark 10M). The background spectrum was acquired using Milli-Q water. For analysis, 150 μL of Au@Ag NPs solution was added to a 96-well transparent plate and measured in the microplate reader within the wavelength range of 300–800 nm.

For transmission electron microscopy (TEM) analysis, samples were deposited onto an ultrathin microgrid (300-mesh carbon support film) and air-dried. Prepared specimens were examined using a TEM (JEM-2100, Japan Electronics) at an accelerating voltage of 200 kV. Distinct fields of view were captured to obtain photographic images, and ImageJ software was used for image analysis to derive relevant data.

For surface-enhanced Raman spectroscopy (SERS) detection, Raman spectra were obtained by a Raman spectrometer (Lab RAM HR, Evolution-HORIBA FRANCE SAS) equipped with a 633 nm helium–neon laser. The laser operated within a power range of 1.6–1.9 mW, with an integration time of 10 s per reading, repeated twice. The spectrometer was calibrated by using the Raman spectrum of an undoped silicon wafer before each experiment.

The SERS substrate was composed of functionalized core–shell microspheres@Au@Ag NPs raspberry-like nanoaggregates, made by mixing a solution of Au@Ag NPs with functionalized core–shell microspheres in a 150:1 volume ratio. Upon the color transformation from red to purple, a specific concentration of analyte solution (4-MBA, R6G, MG, adenine, and uracil) was introduced, bringing the total solution volume to 300 μL. The mixture was incubated before being transferred to a quartz cuvette with a 2 mm optical path for detection. Each sample was measured three times in parallel, and the results were averaged for analysis. In the case of Au NPs and Au@Ag NPs as substrates, the detection method was identical with the above.

Laser power was monitored by using a laser power meter (THORLABS PM100A) after each experiment. The resulting signal intensity was converted into data in units of mW^–1^·s^–1^. Data were processed using the asymmetric least-squares (ALS) technique for baseline correction, and a calibration curve was generated based on characteristic peak intensities.

## Results and Discussion

### Synthesis and Characterization of Au@Ag NPs

**Figure 1:**
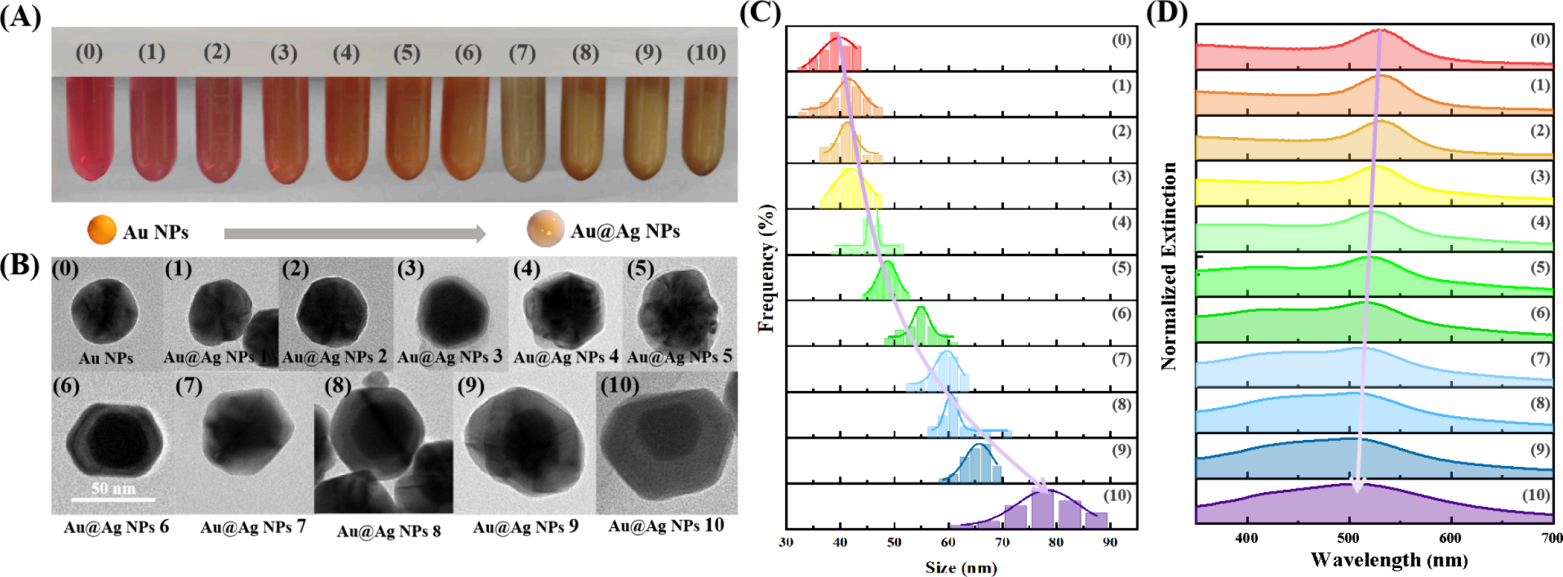
Au NPs and Au@Ag NPs with varied Ag shell thicknesses. (A) Photographs depicting the synthesized Au NPs and Au@Ag NPs with different Ag shell thicknesses, showcasing the solution color transition from red to yellow. (B) Corresponding bright-field TEM images with a scale bar of 50 nm. (C) Particle sizes measured via TEM images using ImageJ software. (D) Normalized UV–vis spectra.

The synthesis of core–shell Au@Ag NPs was carried out through a two-step aqueous reaction. Initially, monodisperse Au NPs were synthesized using a seed growth method, where HAuCl_4_ was reduced by sodium citrate, as described by Bastús et al.^27^ The nanoparticle size was controlled by varying the amount of HAuCl_4_ injected into the solution. UV–vis spectroscopy was employed to determine the sizes of the nanoparticles, which yielded Au NPs with diameters of 30, 40, and 50 nm, stabilized by sodium citrate.^28^ Subsequently, a Ag shell was coated onto the Au core. The lattice compatibility between Ag and Au facilitated the preferential growth of Ag on the Au cores, resulting in the formation of core–shell Au@Ag NPs. The transition of solution color from red to yellow with increasing thickness of the Ag shell (Figure 1A) indicated successful synthesis. The structural dimensions of the nanoparticles were characterized by TEM (Figure 1B). Owing to the image contrast arising from the electron density difference between Au and Ag,^29^ the Au cores and the Ag shells can be easily distinguished in the bright-field TEM images, with darker regions representing the Au cores and semitransparent areas corresponding to the Ag shell. The elemental composition and distribution were further verified by X-ray diffraction (XRD) analysis and elemental mapping using scanning transmission electron microscopy–energy dispersive X-ray spectroscopy (STEM-EDS) (Figures S1 and S2).

In line with our expectation, the thickness of the Ag shell gradually increased with the addition of AgNO_3_. For instance, in the case of the 40 nm Au core, the Ag shell thickness grew from 1.0 nm (Au@Ag NPs 1, Figure 1B(1)) to 19.5 nm (Au@Ag NPs 10, Figure 1B(10)), as measured using ImageJ software based on TEM images. The images revealed that when the shell was relatively thin, the Au@Ag NPs maintained a consistently uniform spherical shape (Figure 1B). However, as the shell thickness increased up to a certain point (Au@Ag NPs 6), a noticeable change in the shape of the Au@Ag NPs was observed, shifting from spherical to a slightly elliptical form, which was related to the adsorption capacity of sodium citrate on different crystalline surfaces. An associated histogram, derived from TEM image analysis, illustrated the frequency distribution of particle sizes (Figure 1C), indicating a gradual increase in the particle size with the addition of AgNO_3_ and sodium citrate. The size of the Au core particles was determined using a previously established formula,^28^ and the thickness of the Ag shell layer was measured using ImageJ software on TEM images.

The colors exhibited by noble metal nanoparticles are indicative of their selective extinction of light across various wavelengths, corresponding to the complementary colors of the absorbed light. The changes in UV–vis spectra during the growth process are depicted in Figure 1D, presented in a normalized format to highlight the blue-shift phenomenon from 530 to 500 nm as the thickness of the Ag shell layer increases. This blue shift in the LSPR peak primarily results from symmetric coupling between the Au–Ag interface and the external Ag shell.^30,31^ Additionally, the appearance of a Ag peak at around 400 nm confirms the successful synthesis of Au@Ag NPs.

### Optimization of Au@Ag NPs and Functionalized PS Core–Shell Microspheres for Raspberry-Like Nanoaggregate Synthesis

Core–shell Au@Ag NPs typically produce stronger SERS signals compared to Au or Ag NPs alone as SERS substrates, owing to the enhanced electromagnetic field of the Au core amplifying the Raman signals on the Ag shell surface. However, it is crucial to consider that the shell thickness and core size in this core–shell structure can significantly influence the SERS enhancement. Consequently, we aimed to determine the optimal shell thickness and core size of Au@Ag NPs for achieving the highest SERS enhancement in subsequent experiments.

**Figure 2:**
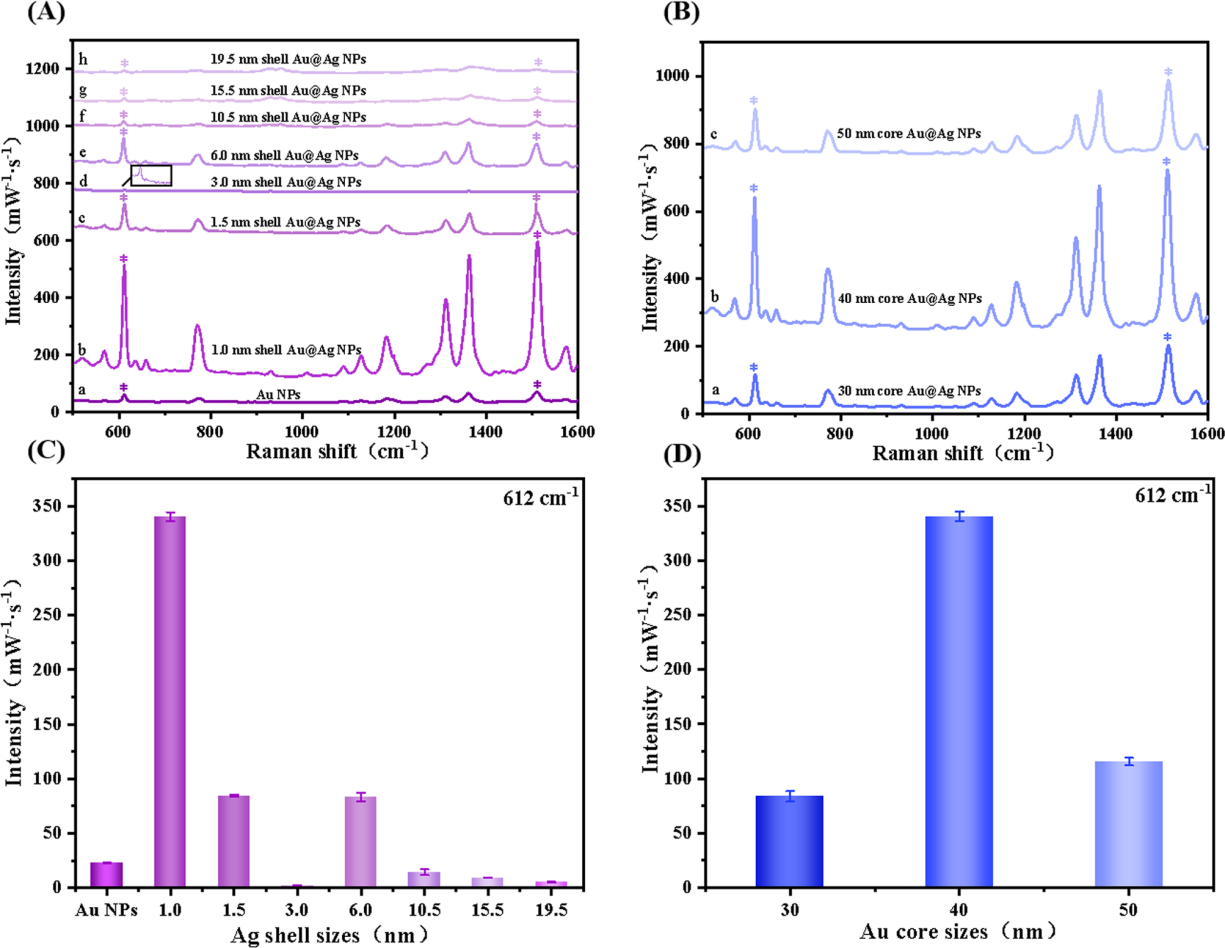
(A) SERS spectra illustrating the core–shell structure of Au@Ag NPs with a 40 nm Au core and varying Ag shell thicknesses (ranging from 1.0 to 19.5 nm). (B) SERS spectra of ultrathin Au@Ag NPs (1.0 nm shell) with different Au core sizes. Variation of the SERS intensity at 612 cm^–1^ for Au@Ag NPs with different (C) Ag shell thicknesses and (D) Au core sizes. Analyte: 1 μg/mL R6G. Excitation wavelength: 633 nm. The concentration of Au NPs used to grow the Ag shell layer was calculated to be 9.22 × 10^–11^ mol/L.

Since nanoparticles with larger particle size are more likely to aggregate, Au@Ag NPs with a 40 nm Au core and different Ag shell thicknesses (from 1.0 to 19.5 nm) were selected to analyze the variation in their Raman signals. We performed SERS analysis using 1 μg/mL of R6G as the target molecule and 633 nm laser excitation. As shown in Figure 2A, the SERS enhancement of Au@Ag NPs exhibited significant dependence on the thickness of the Ag shell. Specifically, 1.0 nm shell Au@Ag NPs exhibited significantly amplified signal intensity compared to uncoated Au NPs (with a diameter of 40 nm) (Figure 2A, spectra a and b). This enhancement can be attributed to the electron ligand effect and local field enhancement within bimetallic nanoparticles.^16^ In other words, the electron cloud density of the atoms on the surface of the Ag shell layer increases, enhancing the interaction between the Ag shell layer and adsorbed molecules, which contributes to the Raman scattering signal enhancement. Furthermore, the coupling strength between the Au core and the Ag shell is maximized, further enhancing the LSPR effect.^32^ With increasing Ag content, the resonance intensity of the Ag shell weakened under the influence of 633 nm laser excitation. At this point, the imaginary components of the dielectric constant of Au@Ag NPs are dominated by the Au cores, resulting in a decrease in SERS activity. According to the results of classical electrodynamics, metal nanoparticles with smaller imaginary components exhibit better SERS activity.^29^ However, upon reaching a specific shell thickness (>5.0 nm shell Au@Ag NPs), the distinctive feature of Ag plasmon gradually became more dominant, resulting in a noticeable increase in resonance intensity, and the SERS intensity increased again. As a result, the optical properties of Au@Ag NPs aligned more closely with those of Ag NPs, leading to the complete shielding of the localized surface plasmon resonance (LSPR) of the Au core (Figure 2A, spectrum e).^33,34^ Further increase in shell thickness may make the Au@Ag NPs more prone to aggregation and precipitation due to the stronger van der Waals interactions, leading to a reduction in SERS signals (Figure 2A, spectra f–h).^35^ In conclusion, Au@Ag NPs with a 1.0 nm shell thickness exhibit the most favorable SERS performance compared to that of other NPs with thicker Ag shell layers.

Moreover, as reported in a previous study,^36^ the amplification of SERS signals is influenced by the size of the metal core. The SERS effect of nanoparticles depends on surface scattering and radiation damping; larger nanoparticles induce stronger enhancement when excited by longer wavelength lasers (e.g., 633 nm), but the increase in particle size exacerbates radiation damping.^37^ To explore the impact of different Au core sizes on the Raman signals of ultrathin silver-shell Au@Ag NPs, evaluations were carried out using varied Au core sizes while maintaining a constant Ag shell thickness. The results revealed that the most prominent SERS signal manifested when the Au core size was 40 nm. This is because Au@Ag NPs with a 40 nm Au core size exhibited a more robust surface scattering effect compared with those with a 30 nm Au core size. In contrast, the radiation damping effect was less pronounced than that of Au@Ag NPs with a 50 nm Au core size (Figure 2B).

**Figure 3:**
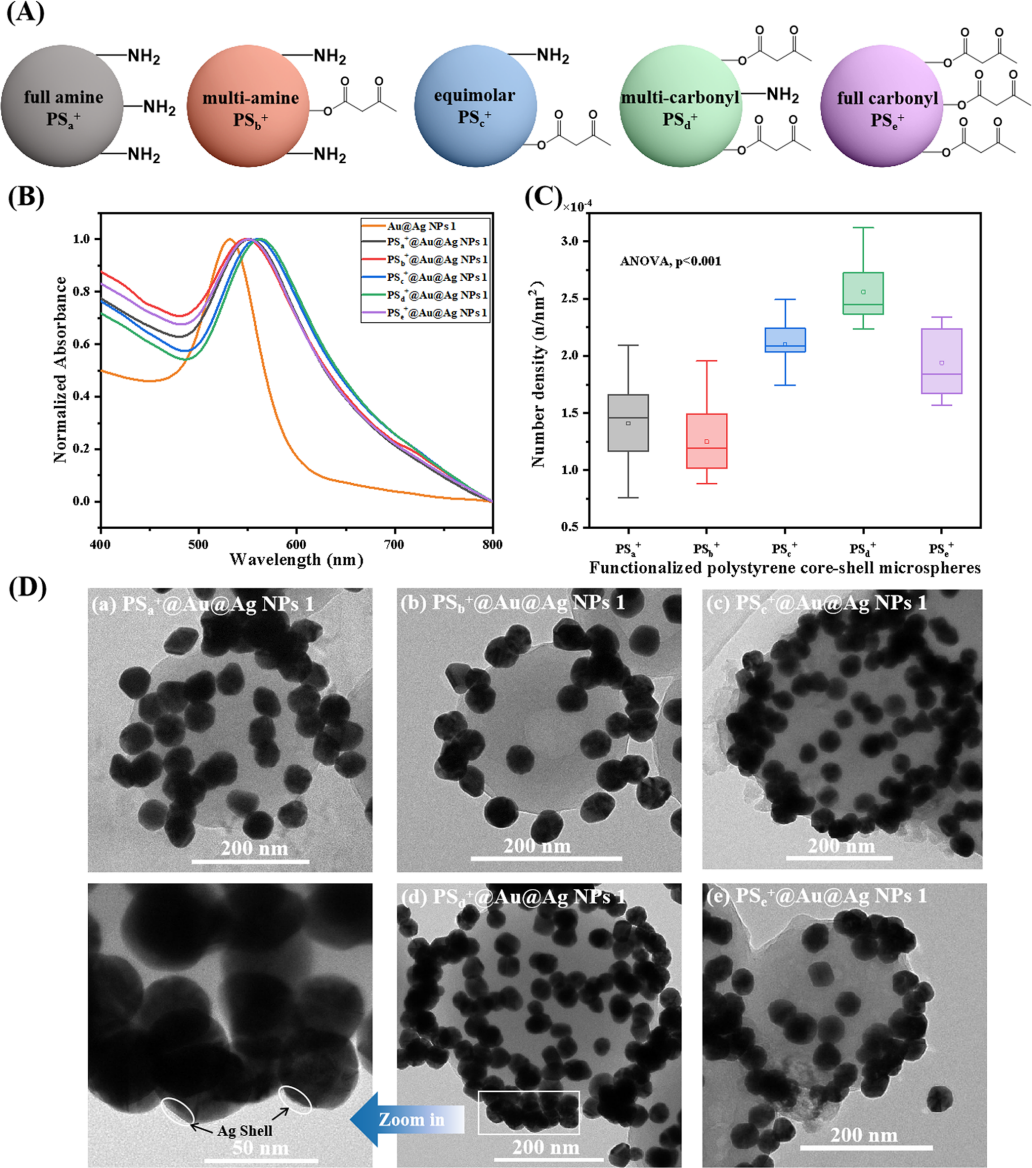
(A) Schematic of surface functional groups on five functionalized PS microspheres. (B) Normalized UV–vis spectra illustrating the combination of five core–shell microspheres with ultrathin Au@Ag NPs (Au core, 40 nm; Ag shell thickness, 1.0 nm). (C) Quantification of Au@Ag NPs number density (n/nm^2^) per core–shell microsphere, ANOVA: *p* < 0.001. (D) TEM images showcasing the formation of raspberry-like nanoaggregates.

The optimal configuration for achieving the highest SERS enhancement was identified from the analysis presented in Figures 2C and 2D, where ultrathin Au@Ag NPs with a 1.0 nm Ag shell and a 40 nm Au core exhibited the most significant enhancement in analyte-induced aggregation. We hypothesize that the enhanced LSPR in the Au@Ag NPs with an ultrathin shell (1.0 nm) also exhibits stronger interparticle plasmonic coupling upon formation of nanoaggregates templated by PS microspheres. Thus, we selected 1.0 nm shell Au@Ag NPs 1 to form raspberry-like nanoaggregates via aqueous self-assembly using five positively charged, surface-functionalized PS core–shell microspheres (full amine core–shell microspheres (PS_a_^+^), multiamine core–shell microspheres (PS_b_^+^), equimolar core–shell microspheres (PS_c_^+^), multicarbonyl core–shell microspheres (PS_d_^+^), and full carbonyl core–shell microspheres (PS_e_^+^) as templates (Figure 3A). Upon introduction of the core–shell microspheres, a noticeable red shift in the LSPR of Au@Ag NPs was observed (Figure 3B). This shift indicated plasmonic coupling between adjacent Au@Ag NPs on the surface of the microspheres. Notably, the most significant red shift was observed with microspheres carrying positive charges and multiple carbonyl groups (PS_d_^+^), indicating their efficient binding through electrostatic interactions with the negatively charged Au@Ag NPs 1 (1.0 nm shell). Indeed, the formation of raspberry-like nanoaggregates was confirmed by TEM for all PS microsphere samples (Figure 3D).

Quantification of the number density of Au@Ag NPs on five microspheres was conducted by counting the nanoparticles on each microsphere (Figure 3C). The results indicate that PS_d_^+^ microspheres exhibited a higher binding capacity for Au@Ag NPs. A detailed examination of the binding interaction between PS_d_^+^ microspheres and Au@Ag NPs 1 revealed the distinct visibility of the Au@Ag NPs on the microsphere surface, with a coverage of about 38% (Figure 3D (d)). To further demonstrate that all of the prepared Au@Ag NPs can bind to the surface of PS_d_^+^ microspheres with high coverage, groups of Au@Ag NP 1, 3, 4, 5, 7, and 9 with different Ag shell layer thicknesses were selected for binding with PS_d_^+^, and it can be seen in Figure S3 that all the binding efficiencies are high and able to produce raspberry-like nanoaggregates.

It is noted that all PS microspheres are positively charged, as verified by their zeta potential data (Figure S4), and therefore, the main driving force of the binding between negatively charged, citrate-protected Au@Ag NPs and positively charged PS microspheres is hypothesized to be electrostatic interactions. Interestingly, however, the microspheres with the most positive zeta potential (PS_a_^+^) did not form the raspberry-like nanoaggregates with the highest surface coverage. In the case of PS_a_^+^, the sample quickly precipitated upon mixing with Au@Ag NPs, owing to the strong electrostatic interactions between the NPs and the microspheres that lead to rapid cross-linking, analogous to diffusion-limited colloidal aggregation (DLCA) kinetics.^38^ In contrast, in the case of PS_d_^+^, which exhibits a smaller positive zeta potential, the formation of nanoaggregates follows slower kinetics, analogous to that of reaction-limited colloidal aggregation (RLCA), resulting in a more compact aggregate morphology. The neutral carbonyl groups on the surface of PS_d_^+^ can also bind to the Au@Ag NPs, similar to the case of cucurbituril-Au@Ag NPs nanocomposites,^39^ further enhancing the surface coverage of NPs on microspheres.

### Investigation of Raman Performance in Multi-Carbonyl Core–Shell Microspheres (PS_d_^+^)@Au@Ag NPs Raspberry-Like Nanoaggregates

Conventional Raman signal detection was performed on a pure 1000 μg/mL R6G solution without any SERS substrate, whereas SERS detection involved a 1 μg/mL R6G solution in the presence of PS_d_^+^@Au@Ag NPs as the SERS substrate. The results depicted in Figure S5 showed minimal intensity in the regular Raman signal from the high-concentration R6G. Conversely, spectra generated by the raspberry-like nanoaggregates for the low-concentration R6G displayed distinct features with sharp and enhanced peaks. Specifically, the peak intensities at 612 and 1512 cm^–1^, representing the two primary peaks of R6G molecule, were analyzed. The enhancement factors (EF) for the PS_d_^+^@Au@Ag NPs were 5.40 × 10^5^ and 5.17 × 10^5^ at 612 and 1512 cm^–1^, respectively (data analysis of EF is in the Supporting Information), indicating the robust SERS capability of the raspberry-like nanoaggregates.

**Figure 4:**
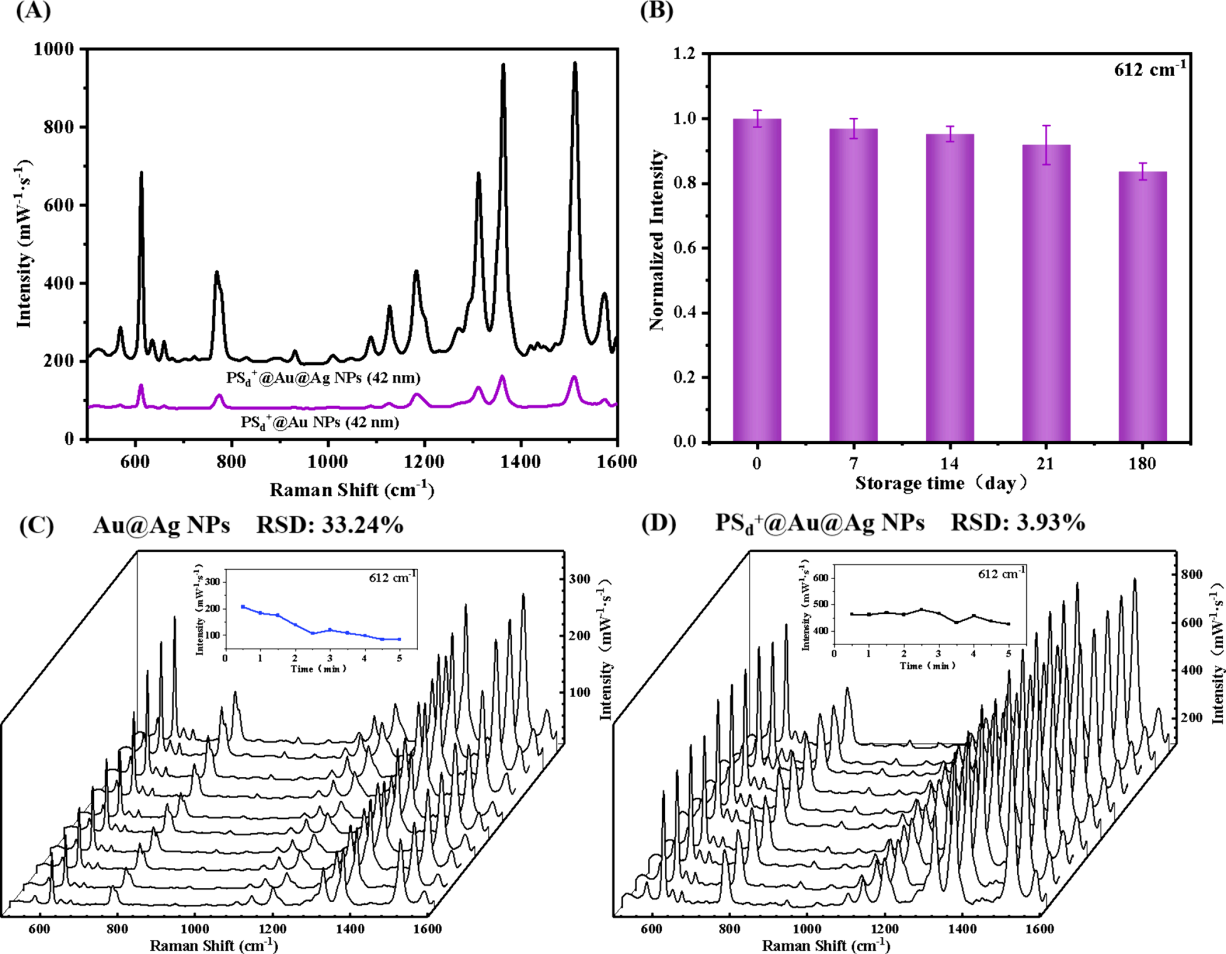
(A) SERS spectra obtained from substrates of PS_d_^+^ @Au@Ag NPs and PS_d_^+^ @Au NPs with 1 μg/mL of R6G. (B) Variation in the SERS band intensity of R6G detected through rapid self-assembly of Au@Ag NPs at various storage times with PS_d_^+^ microspheres. (C and D) Comparative analysis of spectral reproducibility between Au@Ag NPs and PS_d_^+^@Au@Ag NPs, showing consistent results at 10 random positions within the same sample solution.

To evaluate the enhancement capabilities of Au NPs alone versus bimetallic Au@Ag NPs, Au NPs were combined with five different types of PS microspheres, and PS_d_^+^@Au NPs were selected for comparison against PS_d_^+^@Au@Ag NPs for SERS detection of R6G. The results demonstrated that the additional enhancement provided by the ultrathin Ag shell, compared to uncoated Au NPs, also translates to the nanoaggregate form (Figure 4A). Specifically, the PS_d_^+^@Au@Ag NPs consisted of a 40 nm Au core with a 1.0 nm Ag shell, while the PS_d_^+^@Au NPs had a 42 nm Au core, with both being assembled into raspberry-like nanoaggregates under equivalent conditions. As presented in Figure 4A, the PS_d_^+^@Au@Ag NPs exposed to 1 μg/mL R6G exhibited significantly stronger Raman scattering signals compared to PS_d_^+^@Au NPs. The enhancement factors (EF) for PS_d_^+^@Au NPs were 6.50 × 10^4^ and 5.28 × 10^4^ at the characteristic peaks of 612 and 1512 cm^–1^, respectively. In comparison, the signal enhancement with PS_d_^+^@Au@Ag NPs as the substrate was an order of magnitude higher than that with PS_d_^+^@Au NPs, indicating the potent SERS enhancement effects of the Au@Ag NPs (Figure S5).

To minimize the potential influence of raspberry-like nanoaggregates on the SERS interference, these nanoaggregates were used as a blank control group for SERS analysis. As illustrated in Figure S6, the blank substrate exhibited a nearly negligible SERS signal, confirming that the nanoaggregates do not interfere with the subsequent detection of the analyte.

In addition, the stability of the SERS substrate and the reproducibility of the SERS signal are of paramount importance in practical applications. Au@Ag NPs stored at room temperature for different periods of time were mixed with PS_d_^+^ microspheres for 5 min before being exposed to 1 μg/mL of R6G for immediate SERS detection. As illustrated in Figure 4B, the PS_d_^+^@Au@Ag NPs exhibited a percentage RSD ranging from 2.50% to 6.54% within any specific day, and an RSD of 6.69% over the entire 180-day period. This indicates that the individual components of the raspberry-like nanoaggregates possess excellent long-term storage capabilities and are ready for rapid use. The reproducibility of SERS signals was investigated by using R6G as a probe molecule. In each analysis, 10 data points were randomly collected from various positions across the substrate. As shown in Figures 4C and 4D, employing PS_d_^+^@Au@Ag NPs as a substrate yielded notably consistent SERS spectra, with a relative RSD value of 3.93%. Conversely, employing Au@Ag NPs as substrates resulted in an RSD value of 33.24%, indicating a significantly less reproducible outcome. The detection of R6G on the PS_d_^+^@Au@Ag NPs substrate was performed over several hours, showing stable SERS signals throughout the duration (Figure S7). The enhanced stability and improved spectral reproducibility observed in SERS detection with raspberry-like nanoaggregates can be attributed to their structure formation, which effectively regulates the distribution and density of hotspots to achieve greater uniformity. The raspberry-like configuration, composed of multiple nanoparticles, facilitates the creation of multiple hotspots, thereby mitigating fluctuation in peak intensity within spectra. In contrast, in the presence of analyte molecules, individual nanoparticles are prone to aggregation and sedimentation over time, leading to substrate instability.

### Quantitative Analysis of Trace Wastewater Pollutants and Nucleobases

Initially, attention was focused on 4-mercaptobenzoic acid (4-MBA), a compound with a thiol group that forms a stable linkage with the surface of Au@Ag NPs, commonly employed in SERS sensors and as a Raman reporter.^40,41^ Therefore, the presence of 4-MBA was detected using a PS_d_^+^@Au@Ag NPs substrate with a detection limit of 4 × 10^–9^ M, indicating that the substrate possesses remarkable sensitivity (Figure S8 and Table S2).

To validate the application of the SERS substrate in various contexts, we selected wastewater pollutants and nucleobases for detection, including R6G, MG, adenine, and uracil. In recent years, the discharge of large quantities of industrial wastewater and the misuse of pesticides and veterinary drugs have greatly affected environmental safety, food safety, and human health.^42^ Among these pollutants, organic dyes are considered highly carcinogenic to aquatic flora and fauna.^43^ The increased use of dyes such as R6G and MG in the textile industry or aquaculture has further increased their residues in natural water sources.^44^

**Figure 5:**
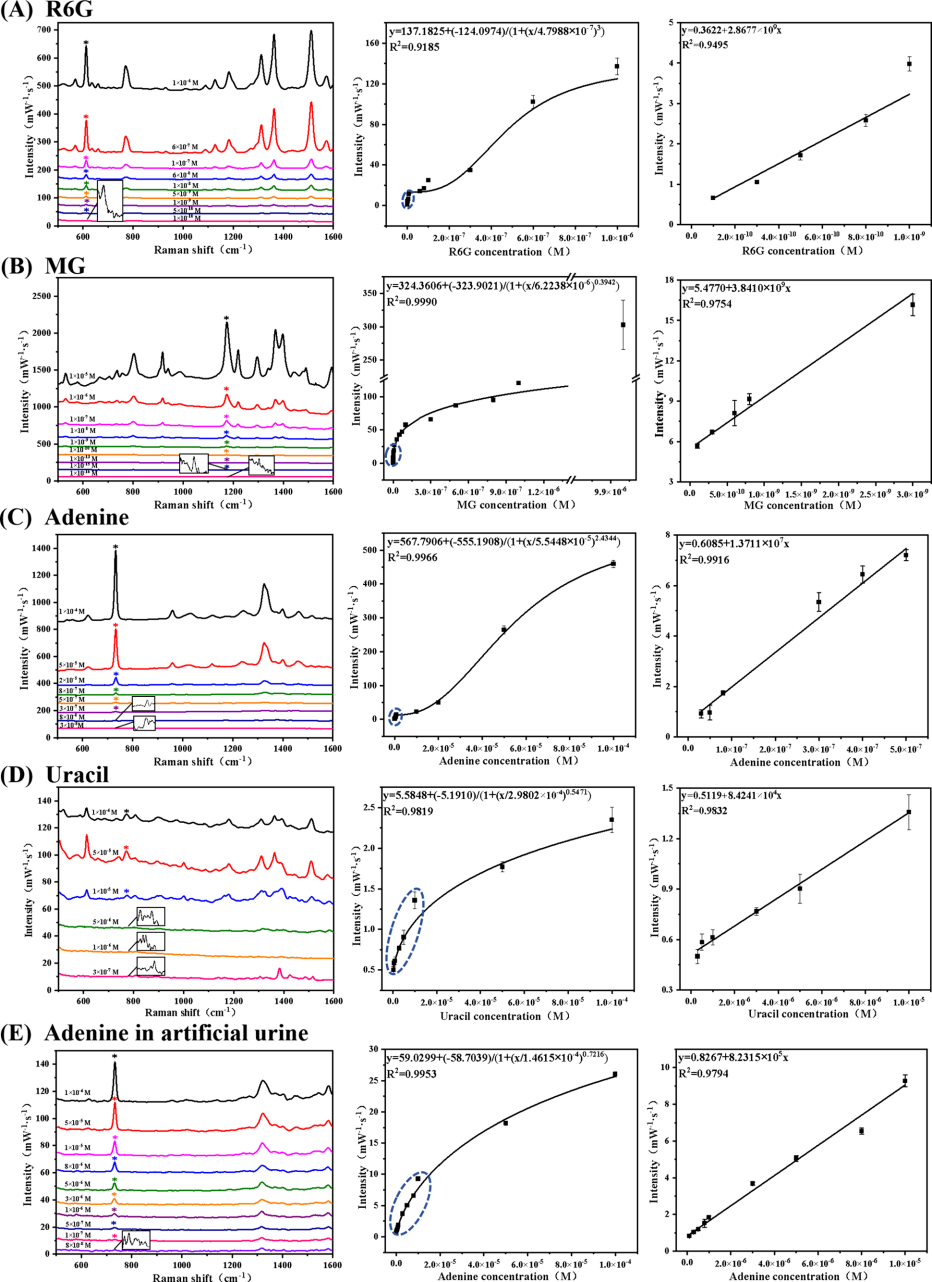
SERS spectra of wastewater pollutants and nucleobase solutions, alongside plots of Raman intensity versus concentration. (A) R6G concentrations ranging from 10^–10^ to 10^–6^ M; (B) MG concentrations ranging from 10^–16^ to 10^–5^ M; (C) adenine concentrations ranging from 3 × 10^–8^ to 10^–4^ M; (D) uracil concentrations ranging from 3 × 10^–7^ to 10^–4^ M; and (E) adenine concentrations in artificial urine ranging from 8 × 10^–8^ to 10^–4^ M.

To verify the feasibility of PS_d_^+^@Au@Ag NPs for the sensitive detection of wastewater pollutants, Raman analyses of the R6G and MG solutions were performed. The Raman spectra of R6G solution and MG powder with characteristic peaks (e.g., 612 and 1173 cm^–1^) are shown in Figures S9 and S10 and Table S1. Detection was performed over a concentration range from 10^–10^ to 10^–6^ M for R6G and 10^–16^ to 10^–5^ M for MG. The constructed calibration curves exhibited robust linear correlations in the lower concentration range and a logarithmic relationship in the higher concentration range (Figures 5A and 5B). The detection limits for R6G and MG were determined to be 1 × 10^–10^ and 1 × 10^–16^ M, respectively. Comparative assessments of MG detection limits within solution-based SERS systems are provided in Tables S3 and S4. Most research papers have utilized solid-based Raman substrates for detection, with detection limits ranging from 10^–6^ to 10^–13^ M for R6G^45−47^ and 10^–7^ to 10^–12^ M for MG.^48−50^ In contrast, liquid-based SERS substrates typically exhibit higher detection limits. Our method, however, allows for direct detection using liquid-based SERS substrates, achieving ultrahigh sensitivity compared to the existing literature.

Subsequently, the SERS capabilities of the raspberry-like nanoaggregates were further demonstrated in the detection of nucleobases, specifically adenine and uracil. These molecules interact intricately with Ag and Au NPs and are utilized in disease detection,^51^ various biomedical applications,^52^ and DNA hybridization.^53^ The detection limits for adenine and uracil were determined to be 3 × 10^–8^ and 3 × 10^–7^ M, respectively (Figures 5C and 5D). The weaker spectral quality of uracil at low concentrations, compared to adenine, may be due to its relatively weak adsorption on the surface of Au@Ag NPs.^54^ Comprehensive analyses of adenine and uracil detection limits within solution-based SERS systems are illustrated in Tables S5 and S6, showcasing the achievement of low detection limits compared to those in other research papers and demonstrating high sensitivity.

During the detection process, a notable phenomenon was observed, where the increments in peak intensity gradually decreased with increasing analyte concentration. This phenomenon is attributed to the saturation of intrinsic hotspots on the Au@Ag NPs surface. At low analyte concentrations, stronger SERS signals were obtained as analytes occupied the spaces between the nanoparticles. However, at higher concentrations, excess analyte molecules could no longer bind at or near the plasmonic hotspots, resulting in a reduced prominence of the SERS effect and leading to a logarithmic relationship across a broader concentration range. Accordingly, the SERS intensities of the substances detected in this study exhibited a logistic logarithmic fit, which has been well-suited to a wide range of concentrations.

Solid-based SERS substrate detection often utilizes the coffee ring effect to construct regular nanoarrays at the solvent evaporation edge.^55^ Although the detection limit is lower than that of the liquid-based substrates, the prepared solid-based substrates often show a large variation in signal intensity at different positions within the coffee ring. Adjusting the solvent and nanoparticle concentration to achieve regular arrays at the edges of evaporated droplets remains a challenge.^56^ In contrast, our PS_d_^+^@Au@Ag NPs substrate can directly detect the liquid sample without any modification, which is convenient and fast and exhibits a good linear relationship within certain concentration intervals, allowing for rapid quantification of the analytes. The detection limits of R6G, MG, adenine, and uracil were determined to be 1 × 10^–10^, 1 × 10^–16^, 3 × 10^–8^, and 3 × 10^–7^ M, respectively, demonstrating comparable or even lower detection limits compared to solid-based substrates and those reported in the literature (Tables S3–S6).

### Quantification of Adenine in Artificial and Clinical Urine Samples

The identification of specific biomarkers is crucial for diseases diagnosis.^57,58^ Elevated levels of adenine in urine have been correlated with conditions such as gout, active leukemia, and various malignant tumors, presenting an opportunity for the early detection of diseases, including lung cancer.^59^

The composition and pH of artificial urine can be precisely controlled, ensuring consistency and reproducibility of each experiment. Accordingly, to explore the practical detection capabilities of raspberry-like PS_d_^+^@Au@Ag NPs nanoaggregates in real-world samples, we prepared spiked samples with varying concentrations of adenine solutions (ranging from 8 × 10^–8^ to 10^–4^ M) using artificial urine. These samples did not require any prior treatment. In particular, the raspberry-like PS_d_^+^@Au@Ag NPs were directly mixed with the adenine-infused urine and subjected to SERS measurements. The intensity variation of the Raman peak at 733 cm^–1^, corresponding to different concentrations of adenine in the urine, is depicted in Figure 5E. Remarkably, this Raman peak remained detectable even at micromolar concentrations, starting from 8 × 10^–8^ M. Further analysis of the data yielded a fitting equation and correlation coefficients (Figure 5E), highlighting the potential practical utility of raspberry-like PS_d_^+^@Au@Ag NPs in the quantitative analyses of real biological samples.

To further demonstrate the matrix tolerance of the raspberry-like PS_d_^+^@Au@Ag NPs nanoaggregates, we attempted to detect adenine in clinical urine samples. Urine from two gout patients and one healthy individual was filtered twice through a 0.22 μm membrane and mixed directly with PS_d_^+^@Au@Ag NPs without dilution. Adenine was successfully detected in the urine of both gout patients but not in the healthy individual (Figure S13). To assess the feasibility of quantitative adenine detection, we employed the standard addition method in a 10-fold diluted urine sample from the healthy individual. As illustrated in Table S7, recoveries ranged from 85.87% to 114.82% with an RSD value of 6.58%. These findings highlight the potential of raspberry-like PS_d_^+^@Au@Ag NPs for noninvasive routine monitoring in personalized healthcare, specifically for the quantitative detection of trace adenine in urine matrices, facilitating highly sensitive and accurate disease detection.

## Conclusion

In conclusion, this study introduces an efficient and straightforward approach for designing and producing raspberry-like plasmonic nanoaggregates, which can be used as SERS substrates for obtaining reproducible spectra. In particular, we demonstrated that highly active and colloidally stable solution-based SERS substrates can be rapidly produced within 5 min via aqueous self-assembly of Au@Ag NPs using positively charged, multicarbonyl-functionalized polystyrene microspheres (PS_d_^+^) as templates, resulting in novel raspberry-like nanoaggregates through electrostatic interactions. Our study reveals key design parameters for raspberry-like plasmonic nanoaggregates, which can be extended to other raspberry-like nanomaterials.

The optimized nanoaggregates can be used as reproducible and stable SERS substrates to rapidly detect a range of wastewater pollutants (e.g., R6G and MG) and nucleobases (e.g., adenine and uracil), with detection limits as low as 1 × 10^–10^, 1 × 10^–16^, 3 × 10^–8^, and 3 × 10^–7^ M, respectively. Furthermore, the nanoaggregates are capable of quantifying spiked adenine in undiluted artificial urine and diluted clinical urine, thereby demonstrating the potential of raspberry-like PS_d_^+^@Au@Ag NPs for the quantitative analysis of real biological samples with clinical applications.

Looking forward, the surface of our nanoaggregates could be further modified to bind specific molecules, forming SERS probes for the selective detection of various substances such as specific DNA hairpins for biomarker detection. Additionally, the hydrophobic properties of PS microspheres offer opportunities for integration with enrichment and detection processes. These versatile applications extend to diverse fields such as sensing, fluorescence detection, and drug release monitoring.

## Supporting Information Available

Reagents and materials; synthesis of surface-functionalized PS core–shell microspheres; data analysis of enhancement factors (EF); X-ray diffraction spectroscopy (XRD) of Au NPs and Au@Ag NPs; energy dispersive X-ray spectroscopy (EDS) of Au NPs and Au@Ag NPs; binding of Au@Ag NPs 1, 3, 4, 5, 7, and 9 to PS_d_^+^ microspheres; zeta potential data of five different functionalized core–shell microspheres; SERS enhancement of PS_d_^+^@Au@Ag NPs and PS_d_^+^@Au NPs; SERS detection of 4-MBA; background SERS signal of PS_d_^+^@Au@Ag NPs; SERS intensity of 1 μg/mL R6G on PS_d_^+^@Au@Ag NPs substrates over time lapsed after SERS sample preparation; Raman spectra of 1000 μg/mL of R6G solution, MG powder, adenine powder, and uracil powder; spectrum of adenine in clinic urine samples; table of Raman band assignments for 4-MBA, R6G, MG, adenine, and uracil; SERS detection of 4-MBA reported in the literature; SERS detection of R6G reported in the literature; SERS detection of MG reported in the literature; SERS detection of adenine reported in the literature; SERS detection of uracil reported in the literature; and detection of adenine in urine of healthy individual based on PS_d_^+^@Au@Ag NPs (PDF)
