# Supplementary material for: Raspberry-Like Plasmonic Nanoaggregates with Programmable Hierarchical Structures for Reproducible SERS Detection of Wastewater Pollutants and Biomarkers
Source: Anal Chem. 2024 Oct 24;96(44):17620–30. doi: 10.1021/acs.analchem.4c03533 (PMC11541892; doi:10.1021/acs.analchem.4c03533)
Supplement: Supplementary file 1 — ac4c03533_si_001.pdf [file ac4c03533_si_001.pdf]

SUPPORTING INFORMATION

Raspberry-like plasmonic nanoaggregates with programmable hierarchical structures for reproducible SERS detection of wastewater pollutants and biomarkers

Huimin Xie,[∇†] Shuyu Zhu,[∇†] Ping Wen,[†] Deyue Zhou,[‡] Yidan Yin,[‡] Yang Lan,[§] Tung-Chun Lee,[*‡] Yuewen Zhang,[*†] and Qiaosheng Pu[†]

[†] College of Chemistry and Chemical Engineering, Lanzhou University, Lanzhou 730000, China

[‡] Institute for Materials Discovery, University College London, London, WC1H 0AJ, U.K.

[§] Department of Chemical Engineering, University College London, London, WC1E 7JE, U.K.

* Email: tungchun.lee@ucl.ac.uk; zhangyw@lzu.edu.cn

[∇]H.X. and [∇]S.Z. contributed equally to this work.

TABLE OF CONTENTS

Reagents and Materials **S3**
Synthesis of Surface-functionalized PS Core-shell Microspheres **S3**
Data Analysis of Enhancement Factor (EF) **S3**
Figure S1 - X-ray Diffraction Spectroscopy (XRD) of Au NPs and Au@Ag NPs **S4**
Figure S2 - Energy Dispersive X-ray Spectroscopy (EDS) of Au NPs and Au@Ag NPs **S5**
Figure S3 - Binding of Au@Ag NPs 1, 3, 4, 5, 7, 9 to PS_d^+ Microspheres **S6**
Figure S4 - Zeta Potential Data of Five Different Functionalized Core-shell Microspheres ..**S7**
Figure S5 - SERS Enhancement of PS_d^+@Au@Ag NPs and PS_d^+@Au NPs **S8**
Figure S6 - Background SERS Signal of PS_d^+@Au@Ag NPs. **S9**
Figure S7 - SERS Intensity of 1 μg/mL R6G on PS_d^+@Au@Ag NPs Substrates over Time Lapsed after SERS Sample Preparation. **S10**
Figure S8 - SERS Detection of 4-MBA. **S11**
Figure S9 - Raman Spectra of 1000 μg/mL of R6G Solution **S12**
Figure S10 - Raman Spectrum of MG Powder **S13**
Figure S11 - Raman Spectrum of Adenine Powder **S14**
Figure S12 - Raman Spectrum of Uracil Powder **S15**
Figure S13 - Raman Spectrum of Adenine in Clinic Urine Samples **S15**
Table S1: Raman Band Assignments for 4-MBA, R6G, MG, Adenine and Uracil **S16**
Table S2: SERS Detection of 4-MBA Reported in the Literature. **S18**
Table S3: SERS Detection of R6G Reported in the Literature. **S19**
Table S4: SERS Detection of MG Reported in the Literature. **S20**
Table S5: SERS Detection of Adenine Reported in the Literature. **S21**
Table S6: SERS Detection of Uracil Reported in the Literature. **S22**
Table S7: Detection of Adenine in Urine of Healthy Individual Based on PS_d^+@Au@Ag NPs **S23**
References **S24**

Reagents and Materials

Gold(III) chloride solution ($HAuCl_4$), silver nitrate ($AgNO_3$), sodium citrate, Malachite Green (MG), adenine, and uracil were purchased from Aladdin Reagent (Shanghai) Co., Ltd. 4-Mercaptobenzoic acid (4-MBA) was procured from Sun Chemical Technology (Shanghai) Co., Ltd. Rhodamine 6G (R6G) was purchased from Merck Chemical Technology (Shanghai) Co., Ltd. Artificial urine was purchased from Tianjin Huasheng Chemical Reagent Co., Ltd. 2-(Methacryloyloxy)ethyl acetoacetate (AAEM) and 2-Aminoethyl methacrylate hydrochloride (AEMH) were purchased from Shanghai Macklin Biochemical Technology Co., Ltd. Positively charged polystyrene (PS) microspheres with five different functionalized groups, including full amine core-shell microspheres (PS_a^+), multi-amine core-shell microspheres (PS_b^+), equimolar core-shell microspheres (PS_c^+), multi-carbonyl core-shell microspheres (PS_d^+), and full carbonyl core-shell microspheres (PS_e^+), were synthesized in our laboratory. Milli-Q water was utilized in all experiments.

Synthesis of Surface-functionalized PS Core-shell Microspheres

Firstly, 0.15 mmol of AEMH and 0.75 mmol of AIBA were dissolved in 150 mL of ultrapure water and degassed under nitrogen for 30 minutes at room temperature. The solution was then heated to 80 °C, and 75 mmol of St was added. After stirring for 24 hours, the mixture was purified by centrifugation and washed three times with ultrapure water. Upon re-dispersion in 150 mL of ultrapure water, DVB, St and different ratios of functionalized monomers (AAEM and AEMH) were added for the cross-linking growth of the shell layer, resulting in the formation of five types of functionalized PS microspheres: full amine core-shell microspheres (PS_a^+), multi-amine core-shell microspheres (PS_b^+), equimolar core-shell microspheres (PS_c^+), multi-carbonyl core-shell microspheres (PS_d^+), and full carbonyl core-shell microspheres (PS_e^+).

Data Analysis of Enhancement Factor (EF)

EF serves as a quantitative measure of the level of Raman scattering enhancement when molecules are adsorbed on a SERS substrate. The EF can be quantified using as following equation:[1]

$$\mathrm{EF} = \frac{I_{SERS}N_{NR}}{I_{NR}N_{SERS}} \quad \text{(Equation 1)}$$

Where I_{SERS} denotes the peak intensity of the surface-enhanced Raman spectra; I_{NR} denotes the peak intensity of conventional Raman (Normal Raman) spectra; N_{SERS} denotes the number of molecules tested by surface-enhanced Raman spectroscopy; and N_{NR} denotes the number of molecules tested by conventional Raman spectroscopy.

Given that the surface-enhanced Raman substrate is in solution, the number of molecules is directly related to the quantity of substance through $N = N_A \times n$. Therefore, substituting C_{SERS} and C_{NR} for N_{SERS} and N_{NR} respectively, the equation can be expressed as

$$\mathrm{EF} = \frac{I_{SERS}C_{NR}}{I_{NR}C_{SERS}} \quad \text{(Equation 2)}$$

Where C_{SERS} represents the solution concentration for surface-enhanced Raman detection; and C_{NR} represents the solution concentration for conventional Raman detection.

Figure S1 - X-ray Diffraction Spectroscopy (XRD) of Au NPs and Au@Ag NPs

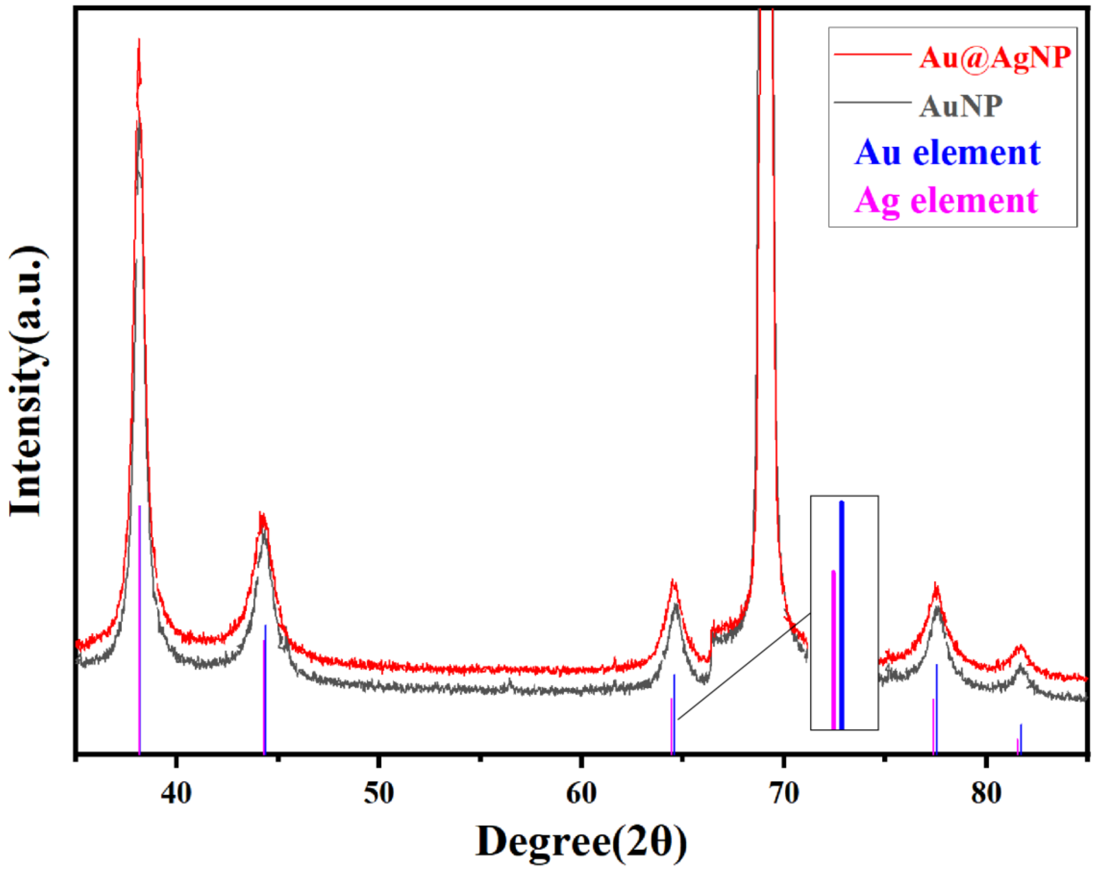

Figure S1. XRD characterisation of AuNPs and Au@Ag NPs; the blue and pink vertical lines are the XRD standard peak positions for gold and silver.

Note for Figure S1: Although Au and Ag are chemically similar, the peak position of Ag appears at 2θ, which is slightly smaller than that of Au. As illustrated in the figure, the blue and pink vertical lines represent the standard diffraction peaks of Au and Ag, respectively. The overall peak position of Ag is slightly earlier than that of Au, confirming the presence of both elements.

Figure S2 - Energy Dispersive X-ray Spectroscopy (EDS) of Au NPs and Au@Ag NPs

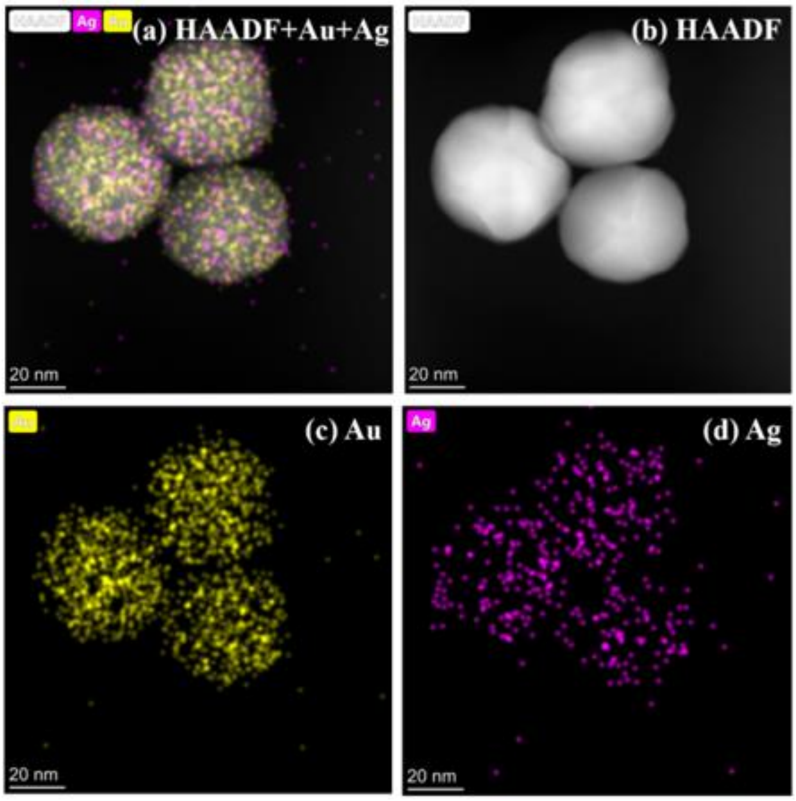

Figure S2. STEM-EDS elemental mapping images of Au@Ag NPs 1. (a) Dark-field image superimposed with Au and Ag elemental distributions; (b) High-angle annular dark-field (HAADF) image of Ag-coated Au; (c) Au elemental distribution; (d) Ag elemental distribution.

Note for Figure S2: From the figure, it can be seen that Au is located in the core while Ag is distributed in the outer layer of Au NPs, confirming the generation of ultra-thin shell Au@Ag NPs (1.0 nm shell).

Figure S3 - Binding of Au@Ag NPs 1, 3, 4, 5, 7, 9 to PS_d^+ Microspheres

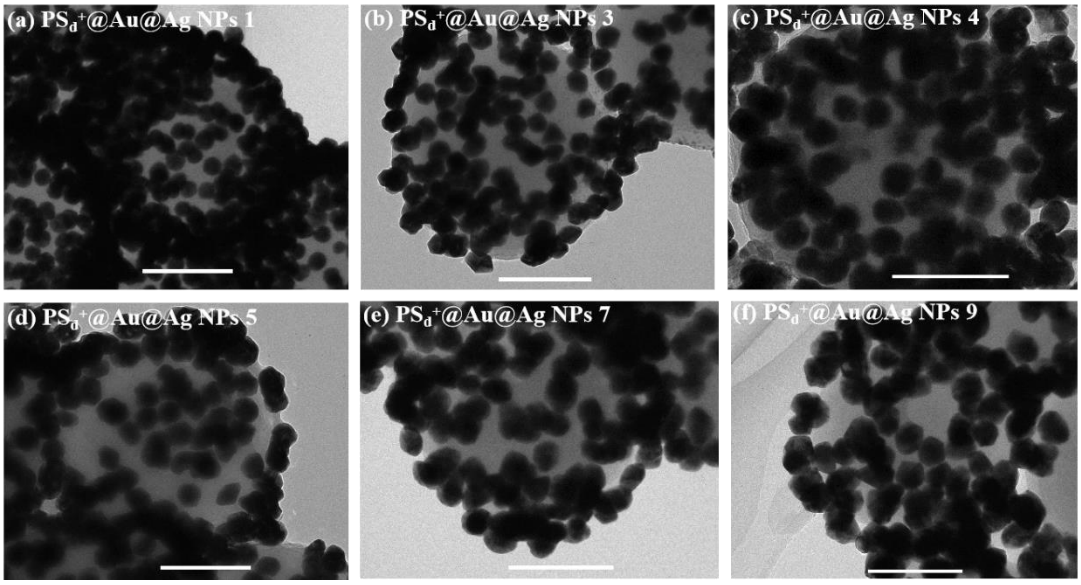

Figure S3. TEM images of Au@Ag NPs 1, 3, 4, 5, 7, 9 with PS_d^+ microspheres to generate a raspberry-like nanoaggregates. Scale bars = 200 nm.

Note for Figure S3: A selection encompassing Au@Ag NPs 1, 3, 4, 5, 7, and 9, each featuring distinct Ag shell thicknesses, was employed for the binding process with PS_d^+ microspheres. The results showcased that the synthesized Au@Ag NPs exhibited a high surface coverage in binding with PS_d^+ microspheres.

Figure S4 - Zeta Potential Data of Five Different Functionalized Core-shell Microspheres

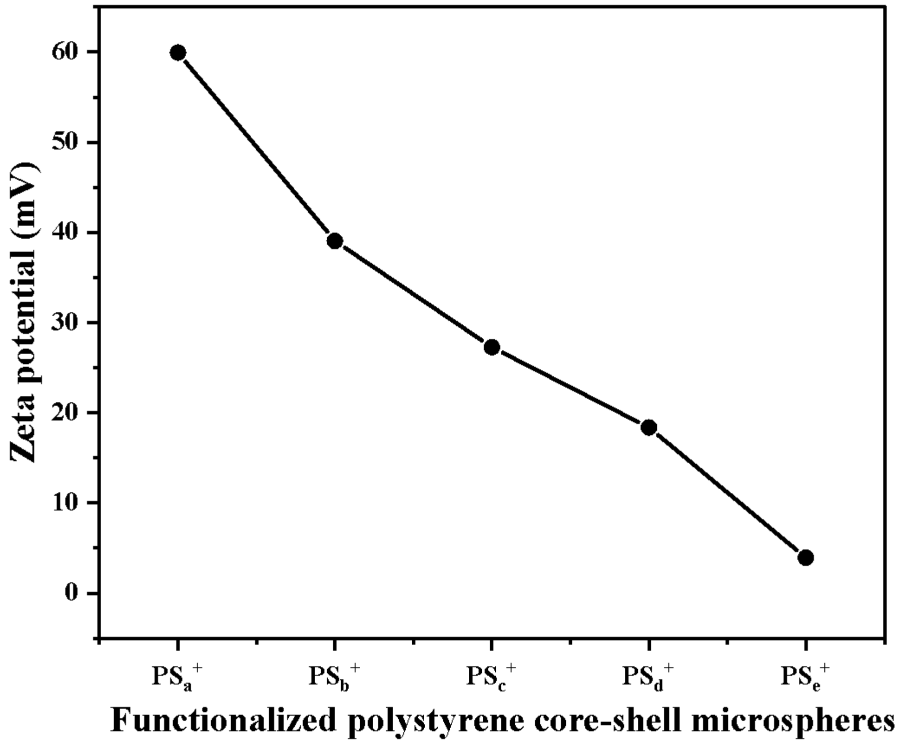

Figure S4. Zeta potential data of five different functionalized core-shell microspheres (full amine core-shell microspheres (PS_a^+), multi-amine core-shell microspheres (PS_b^+), equimolar core-shell microspheres (PS_c^+), multi-carbonyl core-shell microspheres (PS_d^+), and full carbonyl core-shell microspheres (PS_e^+)).

Note for Figure S4: All PS microspheres are positively charged, Therefore, the primary binding mechanism between negatively charged, citrate-protected Au@Ag NPs and positively charged PS microspheres is hypothesized to be electrostatic interactions.

Figure S5 - SERS Enhancement of PS_d^+@Au@Ag NPs and PS_d^+@Au NPs

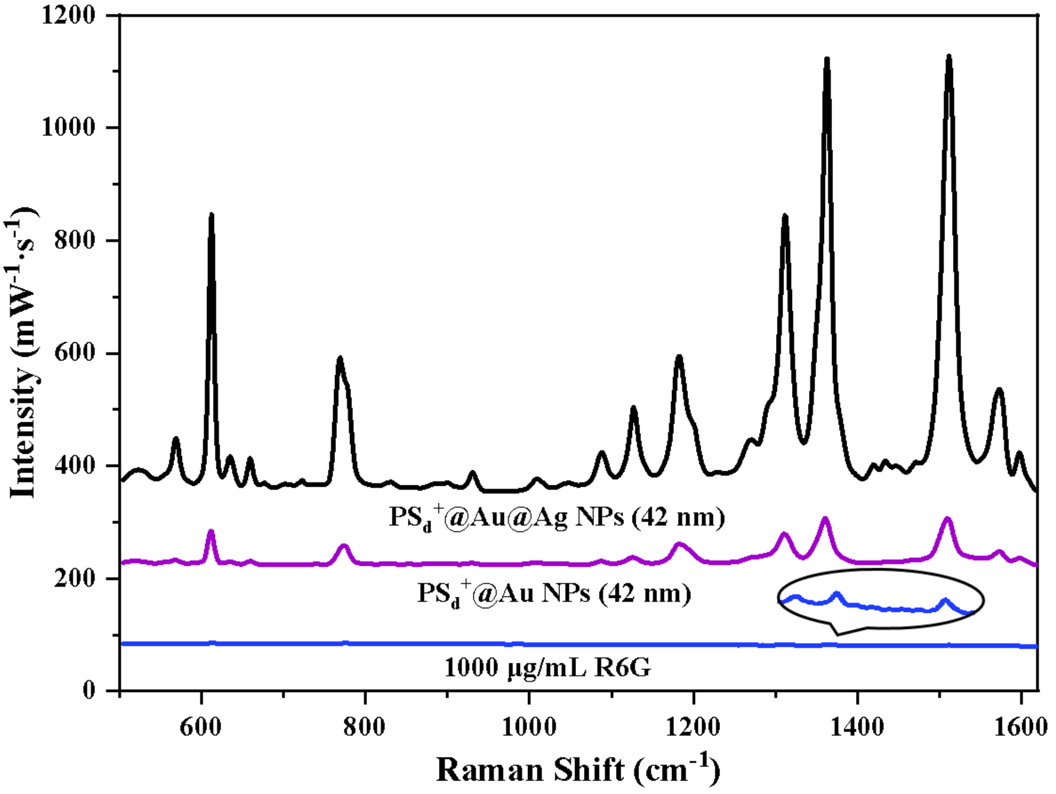

Figure S5. Comparison of the conventional Raman signal of 1000 µg/mL R6G and SERS signal of 1 µg/mL R6G in the presence of PS_d^+@Au@Ag NPs and PS_d^+@Au NPs.

Note for Figure S5: Enhancement factors of the PS_d^+@Au@Ag NPs were computed, yielding values of EF = 5.40×10^5 and 5.17×10^5 at 612 cm^{-1} and 1512 cm^{-1} respectively. Correspondingly, for the 42 nm PS_b^+@Au NPs consisting of the raspberry-like nanoaggregates, the enhancement factors stood at EF = 6.50×10^4 and 5.28×10^4 at these characteristic peaks.

Figure S6 - Background SERS Signal of PS_d^+@Au@Ag NPs

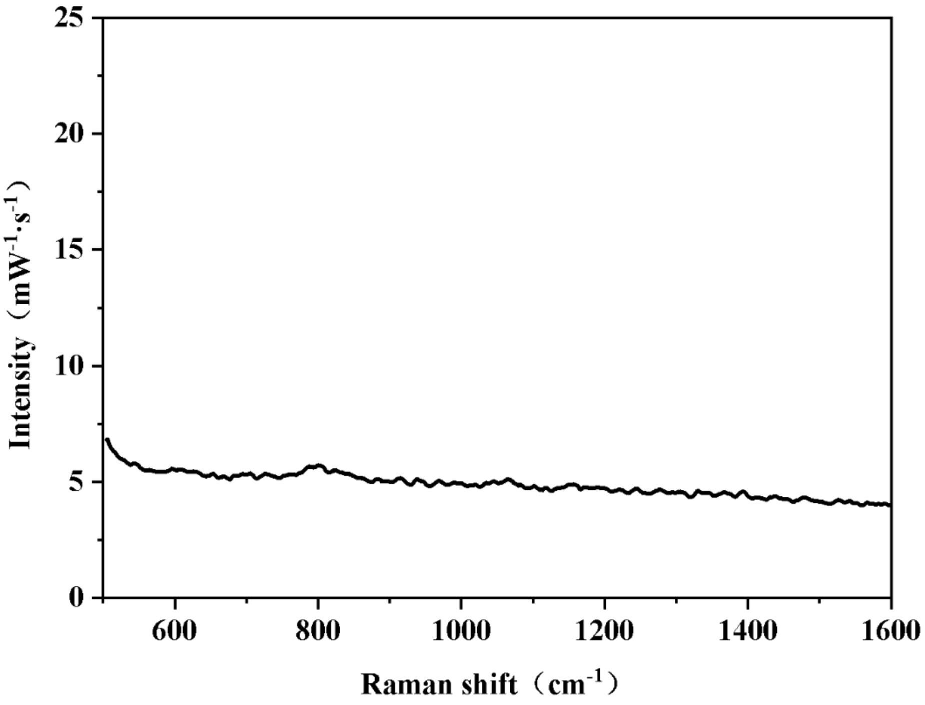

Figure S6. Background SERS signal of PS_d^+@Au@Ag NPs.

Note for Figure S6: To minimize the potential influence of raspberry-like nanoaggregates on SERS interference, these nanoaggregates were used as a blank control group for SERS analysis. As illustrated in Figure S6, the blank substrate exhibited nearly negligible SERS signal, confirming that the nanoaggregates do not interfere with the subsequent detection of the analyte.

Figure S7 - SERS Intensity of 1 μg/mL R6G on PS_d^+@Au@Ag NPs Substrates over Time Lapsed after SERS Sample Preparation

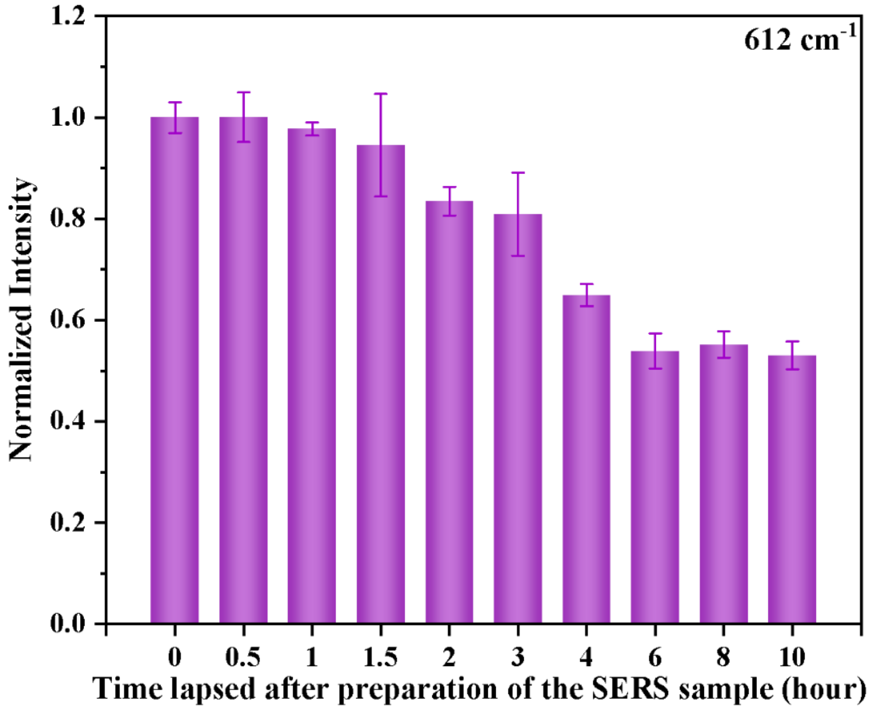

Figure S7. SERS intensity of 1 μg/mL R6G on the PS_d^+@Au@Ag NPs substrates over time lapsed after SERS sample preparation.

Note for Figure S7: The detection of R6G on the PS_d^+@Au@Ag NPs substrate was performed over several hours, during which stable SERS signals were observed for an extended period.

Figure S8 - SERS Detection of 4-MBA.

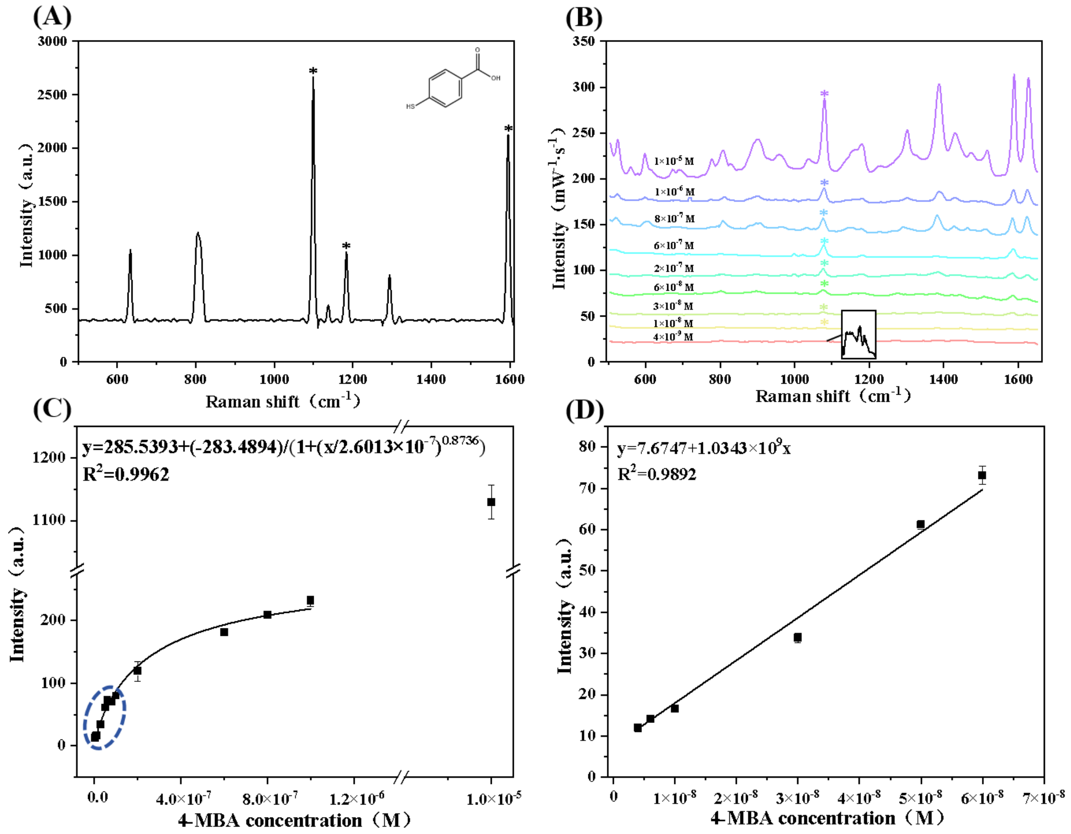

Figure S8. SERS detection of 4-MBA. (A) Raman spectrum of 4-MBA powder; (B) 4-MBA concentrations ranging from 4 × 10^{-9} M to 10^{-6} M; (C) and (D) Plots illustrating the relationship between Raman intensity and concentration.

Note for Figure S8: In the Raman spectrum of 4-MBA powder, the distinct peaks in the 4-MBA powder at 1077 cm^{-1} (C-H deformation vibration), 1181 cm^{-1}, and 1585 cm^{-1} (C–S stretching mode and the breathing mode of the aromatic ring, C = C) were observed (Figure S6A and Table S1). Assessment of 4-MBA solutions, with concentrations ranging from 4×10^{-9} M to 10^{-5} M, exhibited amplified peak intensities at higher concentrations (Figure S8B). The constructed calibration curve based on the peak at 1077 cm^{-1} revealed a commendable linear relationship within the intervals of 4 × 10^{-9} M - 6 × 10^{-8} M, while a logarithmic function fit was observed across a broader concentration span (4 × 10^{-9} M - 10^{-5} M) (Figure S8C and D). Notably, the detection limit reached 4 × 10^{-9} M, enabling comparison with detection limits from solid-based substrate literature sources (Table S2).

Figure S9 - Raman Spectra of 1000 μg/mL of R6G Solution

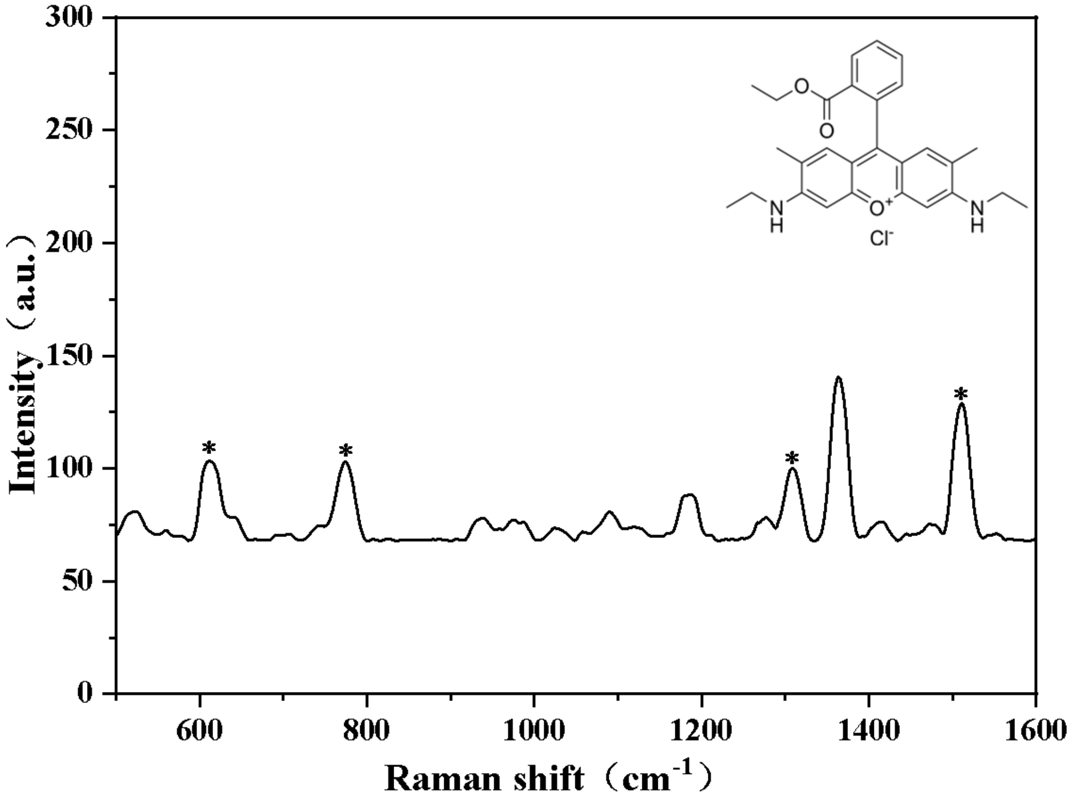

Figure S9. Raman spectra of 1000 μg/mL of R6G solution

Note for Figure S9: The principal Raman bands associated with R6G solution can be attributed to various vibrational modes, including the out-of-plane C–H bending (612 cm^{-1} and 772.4 cm^{-1}), in-plane N–H bending (1313.8 cm^{-1}), and aromatic ring C-C stretching (1511.6 cm^{-1}) modes.

Figure S10 - Raman Spectrum of MG Powder

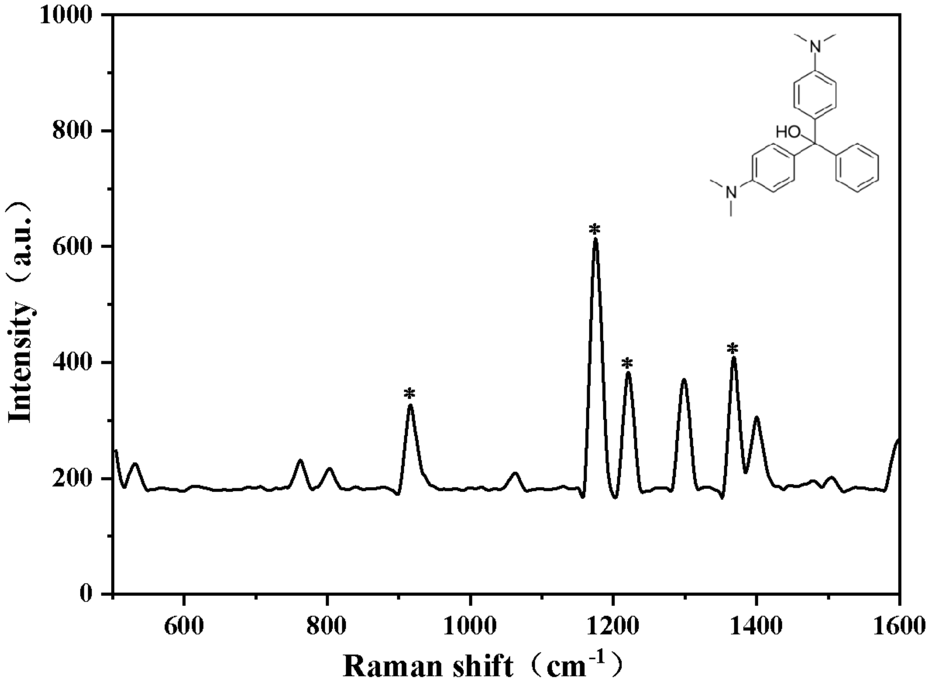

Figure S10. Raman spectrum of MG powder.

Note for Figure S10: We conducted Raman analysis of the powder of MG identifying several characteristic peaks around 917, 1173, 1218, 1367, and 1618 cm^{-1}, consistent with reported literature on MG SERS spectra.

Figure S11 - Raman Spectrum of Adenine Powder

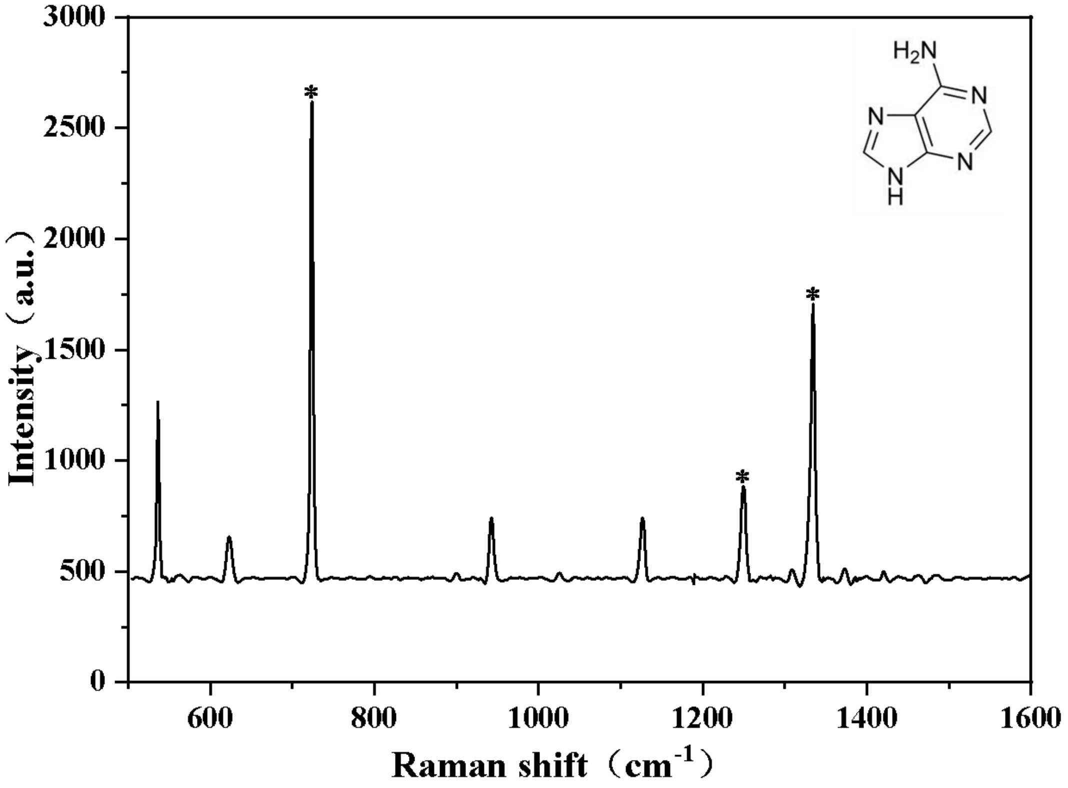

Figure S11. Raman spectrum of adenine powder.

Note for Figure S11: We conducted Raman characterization of adenine powder, revealing distinctive Raman bands attributed to ring breathing vibrations (724 cm^{-1}), in-plane and out-of-plane vibrations of N-H bonds (1251 cm^{-1}), and in-plane stretching vibrations of C-N bonds (1332 cm^{-1}).

Figure S12 - Raman Spectrum of Uracil Powder

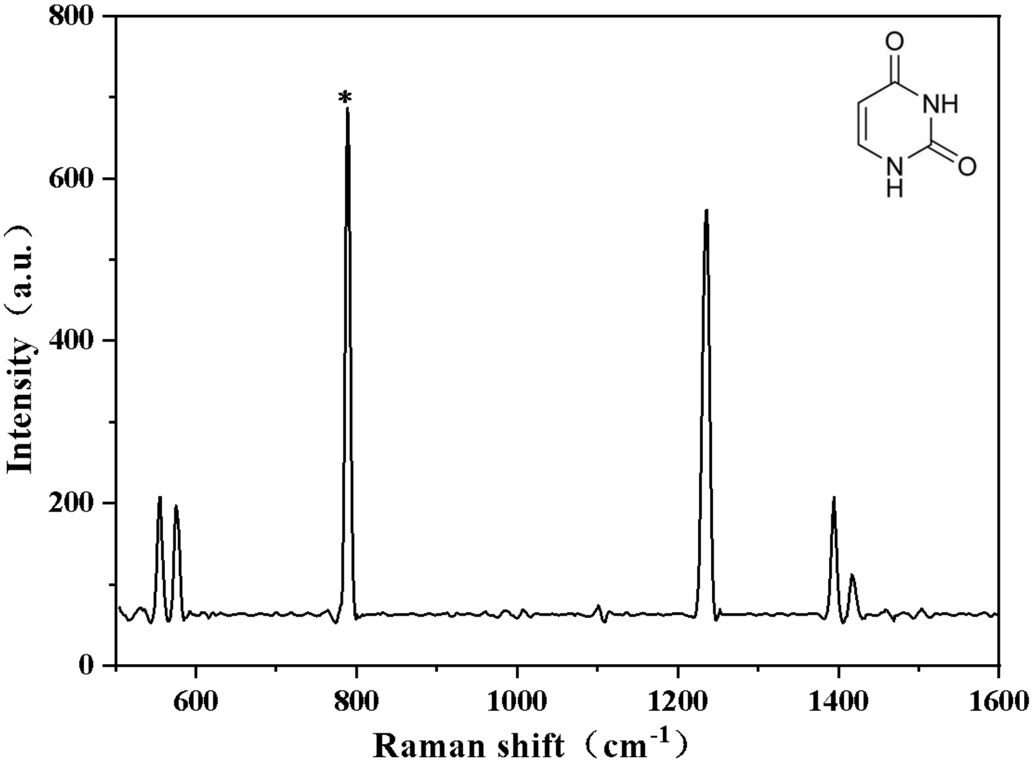

Figure S12. Raman spectrum of Uracil powder.

Note for Figure S12: We conducted Raman characterization of Uracil powder, revealing distinctive Raman bands attributed to ring breath (795 cm^{-1}).

Figure S13 - Raman Spectrum of Adenine in Clinic Urine Samples

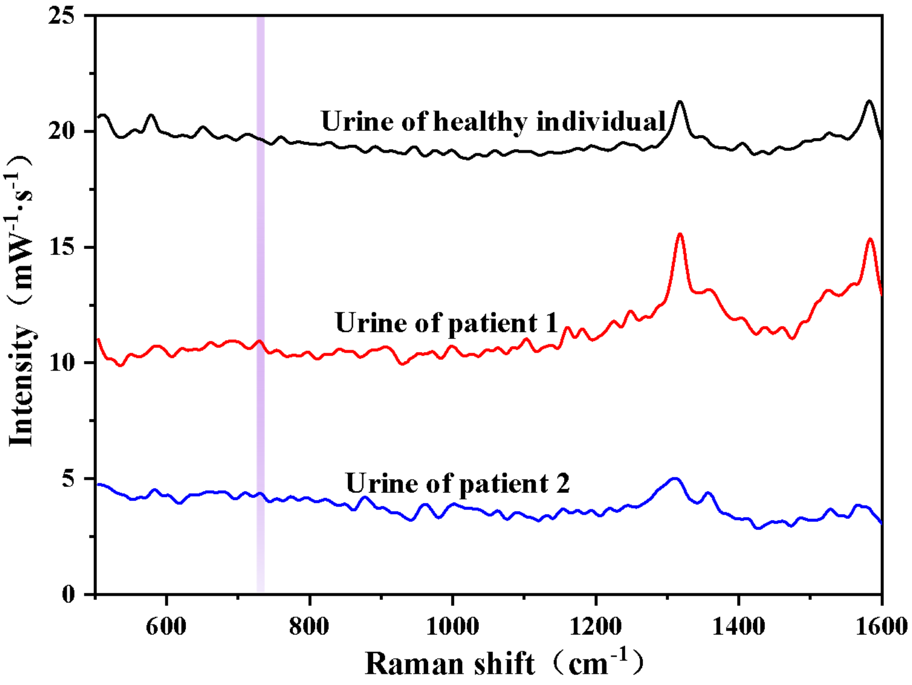

Figure S13. SERS spectrum of adenine (732 cm^{-1}) in clinic urine samples.

Note for Figure S13: Adenine was detected in the urine of both gout patients, but not in the urine of the healthy individual. These findings highlight the potential of raspberry-like PS_d^+@Au@Ag NPs for non-invasive routine monitoring in personalized healthcare, specifically for the quantitative detection of trace adenine in urine matrices, facilitating highly sensitive and accurate disease detection.

Table S1: Raman Band Assignments for 4-MBA, R6G, MG, Adenine and Uracil

Nucleobase	Raman shift (cm^{-1})	Band assignment	Ref
4-MBA	1077	C-H deformation vibration	
4-MBA	1181	C-S stretching mode	2
4-MBA	1585	Aromatic ring breathing mode	
R6G	612	C-H out-of-plane bending	
R6G	772	C-H out-of-plane bending	3
R6G	1314	N-H in-plane bending	
R6G	1512	Aromatic ring C-C stretching	
MG	796	Ring C-H out-of -plane vibrations	
MG	917	Ring skeletal vibrations of radical orientation	
MG	1173	Ring C-H in-plane vibration	4
MG	1218	C-H rocking	
MG	1367	N-Phenyl stretching	
Adenine	724	Ring breathing vibrations	
Adenine	1251	N-H out-of-plane vibrations	5
Adenine	1332	C-N in-plane stretching vibrations	
Uracil	795	Ring breath	5

Table S2: SERS Detection of 4-MBA Reported in the Literature

Substrate type	Substrate material	Limit of detection	Description	Ref
Solid	HyCoS AgPt NPs	10^{-8} M	Formation of hybrid core-shell (HyCoS) AgPt nanoparticles (NPs) by solid state dewetting (SSD) process	6
Solid	FTO/Ag/ZIF-8	10^{-9} M	A series of F-doped SnO_2/Ag/zeolite imidazole framework sandwich structure	7
Solid	Ag-TiO_2	10^{-9} M	Formation of Ag simultaneously deposited and doped TiO_2 hybrid nanoparticles (NPs) by sol–hydrothermal method	8
**Liquid**	**PS_d^+@Au@Ag NPs**	**4×10^{-9} M**	**Raspberry-like nanoaggregates based on multi-carbonyl core-shell microspheres coated with Au@Ag NPs**	**This work**

Note for Table S2： It is noteworthy that the liquid-based SERS substrate achieves a low detection limit for 4-MBA down to 4×10^{-9} M, comparable to that obtained when using solid-based substrates in some cases.

Table S3: SERS Detection of R6G Reported in the Literature

Substrate type	Substrate material	Limit of detection	Description	Ref
Solid	Ag-Au nanoalloy	10^{-8} M	Formation of Ag-Au nanoalloy on silicon substrates by microfluidics	9
Solid	$Ag_{0.5}Au_{0.5}$@P-Si	10^{-9} M	$Au_{0.5}Ag_{0.5}$ alloy nanolayer deposited on pyramidal Si arrays	10
Solid	Ag-Au NPs/MoS_2	10^{-13} M	3D SERS substrate based on Au-Ag bi-metal nanoparticles/MoS2 hybrid with pyramid structure	11
Solid	Ag-Au nanoalloy	10^{-6} M	Preparation of Au:Ag bimetal array on polystyrene (PS) colloidal sphere templates	12
Solid	Au/AgANTs	10^{-9} M	Preparation of highly ordered Au-Ag alloy arrays by electrochemistry	13
**Liquid**	**PS_d^+@Au@Ag NPs**	**10^{-10} M**	**Raspberry-like nanoaggregates based on multi-carbonyl core-shell microspheres coated with Au@Ag NPs**	**This work**

Note for Table S3：It is noteworthy that the liquid-based SERS substrate achieves a detection limit for R6G as low as 10^{-10} M, which is even lower than that obtained using solid-based substrates in some cases.

Table S4: SERS Detection of MG Reported in the Literature

Substrate type	Substrate material	Limit of detection	Description	Ref
Solid	Au-G-AgNs	10^{-10} M	Au NPs-groove-trapped Ag needles	14
Solid	Ag/MoS_2-Ti	10^{-8} M	Ag NPs decorated mesh-like MoS_2 hierarchical nanostructure fabricated on Tifoil	15
Solid	AgNCs	2.62×10^{-7} M	Ag nanocubes of high purification	16
Solid	Ti_3C_2-functionalized Au/Ag	10^{-12} M	Au/Ag bimetallic nanocuboid superlattices coated with Ti_3C_2 nanosheets	17
Liquid	Au/AgANTs	4.07×10^{-12} M	Hollow porous pentagonal Au/Ag alloy nanotubes	18
**Liquid**	**PS_d^+@Au@Ag NPs**	**10^{-16} M**	**Raspberry-like nanoaggregates based on multi-carbonyl core-shell microspheres coated with Au@Ag NPs**	**This work**

Note for Table S4：It is noteworthy that the liquid SERS substrate achieves a lower detection limit for MG, down to 10^{-16} M, which is several orders of magnitude lower than many of the detection limits obtained using solid-based substrates.

Table S5: SERS Detection of Adenine Reported in the Literature

Substrate type	Substrate material	Limit of detection	Description	Ref
Solid	Ag NPs	10^{-11} M	Clusters of silver nanoparticles deposited on three-dimensional copper meshes	19
Solid	Ag/poly(t-butylacrylate)	2×10^{-8} M	Emulsions of Ag nanoparticles encapsulated in poly(t-butylacrylate) (PtBA)	20
Solid	Ag	4.23×10^{-9} M	Ag-grafted sponge	21
Fluid	Ag NPs	10^{-5} M	Citrate-reduced silver nanoparticles	22
Liquid	Ag nanotriangle-based nanopores	10^{-5} M	A silver nanotriangle-based nanopore (diameter < 50 nm) system	23
**Liquid**	**PS_d^+@Au@Ag NPs**	**3×10^{-8} M**	**Raspberry-like nanoaggregates based on multi-carbonyl core-shell microspheres coated with Au@Ag NPs**	**This work**

Note for Table S5：It is noteworthy that the liquid-based SERS substrate achieves a low detection limit for adenine, down to 3×10^{-8} M, comparable to that obtained when using solid-based substrates.

Table S6: SERS Detection of Uracil Reported in the Literature

Substrate type	Substrate material	Limit of detection	Description	Ref
Liquid	Ag NPs	10^{-5} M	Citrate-reduced silver nanoparticles	[22]
Liquid	Au NPs and Ag NPs	10^{-3} M	Citrate-reduced silver nanoparticles	[24]
Liquid	Au@Ag NPs + CB7	10^{-8} M	Self-assembly of core@shell gold@silver nanoparticles (Au@Ag NPs) with cucurbit[7]uril (CB7)	[5]
**Liquid**	**PS_d^+@Au@Ag NPs**	**3×10^{-7} M**	**Raspberry-like nanoaggregates based on multi-carbonyl core-shell microspheres coated with Au@Ag NPs**	**This work**

Note for Table S6: It is noteworthy that the liquid-based SERS substrate achieves a low detection limit for uracil, down to 3×10^{-7} M, which is comparable to or lower than other reports in the literature.

Table S7: Detection of Adenine in Urine of Healthy Individual Based on PS_d^+@Au@Ag NPs

Samples	Spiked (μM)	Found (μM)	Found (%)	RSD (%)
Urine of the healthy individual	1.00	1.08	108.14	3.00
	3.00	3.04	101.17	2.80
	5.00	4.89	97.87	6.58
	8.00	6.87	85.87	3.90
	10.00	11.48	114.82	3.43
	50.00	44.31	88.62	0.18

Note for Table S7: The recoveries for this protocol ranged from 85.87% to 114.82%, with an RSD value of 6.58%. This evidence supports the assertion that raspberry-like PS_d^+@Au@Ag NPs can be used for the quantitative detection of trace adenine in urine matrices.

References

(1) Le Ru, E. C.; Blackie, E.; Meyer, M.; Etchegoin, P. G. Surface Enhanced Raman Scattering Enhancement Factors: A Comprehensive Study. *J. Phys. Chem. C* **2007**, *111* (37), 13794–13803.

(2) Wang, F.; Widejko, R. G.; Yang, Z.; Nguyen, K. T.; Chen, H.; Fernando, L. P.; Christensen, K. A.; Anker, J. N. Surface-Enhanced Raman Scattering Detection of pH with Silica-Encapsulated 4-Mercaptobenzoic Acid-Functionalized Silver Nanoparticles. *Anal. Chem.* **2012**, *84* (18), 8013–8019.

(3) Zaleski, S.; Cardinal, M. F.; Chulhai, D. V.; Wilson, A. J.; Willets, K. A.; Jensen, L.; Van Duyne, R. P. Toward Monitoring Electrochemical Reactions with Dual-Wavelength SERS: Characterization of Rhodamine 6G (R6G) Neutral Radical Species and Covalent Tethering of R6G to Silver Nanoparticles. *J. Phys. Chem. C* **2016**, *120* (43), 24982–24991.

(4) Huang, J.; Ma, D.; Chen, F.; Chen, D.; Bai, M.; Xu, K.; Zhao, Y. Green in Situ Synthesis of Clean 3D Chestnutlike Ag/WO3–x Nanostructures for Highly Efficient, Recyclable and Sensitive SERS Sensing. *ACS Appl. Mater. Interfaces* **2017**, *9* (8), 7436–7446.

(5) Davison, G.; Jones, T.; Liu, J.; Kim, J.; Yin, Y.; Kim, D.; Chio, W.-I. K.; Parkin, I. P.; Jeong, H.-H.; Lee, T.-C. Computer-Aided Design and Analysis of Spectrally Aligned Hybrid Plasmonic Nanojunctions for SERS Detection of Nucleobases. *Adv. Mater. Technol.* **2023** *8* (7), 2201400.

(6) Lin, S.; Habib, M. A.; Burse, S.; Mandavkar, R.; Joni, M. H.; Kunwar, S.; Lee, J. Plasmonic Hybrid Core-Shell (HyCoS) AgPt NP Template Hybridized with GQDs for SERS Enhancement of 4-MBA and BT. *J. Alloys Compd.* **2023**, *952*, 169952.

(7) Xue, X.; Chen, L.; Zhao, C.; Qiao, Y.; Wang, J.; Shi, J.; Lin, Y.; Chang, L. Tailored FTO/Ag/ZIF-8 Structure as SERS Substrate for Ultrasensitive Detection. *Spectrochim. Acta, Part A* **2022**, *282*, 121693.

(8) Yang, L.; Sang, Q.; Du, J.; Yang, M.; Li, X.; Shen, Y.; Han, X.; Jiang, X.; Zhao, B. A Ag Synchronously Deposited and Doped TiO_2 Hybrid as an Ultrasensitive SERS Substrate: A Multifunctional Platform for SERS Detection and Photocatalytic Degradation. *Phys. Chem. Chem. Phys.* **2018**, *20* (22), 15149–15157.

(9) Lin, L.; Li, X.; Gao, H.; Xu, H.; Starostin, S. A.; Ostrikov, K. K.; Hessel, V. Microfluidic Plasma-Based Continuous and Tunable Synthesis of Ag–Au Nanoparticles and Their SERS Properties. *Ind. Eng. Chem. Res.* **2022**, *61* (5), 2183–2194.

(10) Shinki; Sarkar, S. Au0.5Ag0.5 Alloy Nanolayer Deposited on Pyramidal Si Arrays as Substrates for Surface-Enhanced Raman Spectroscopy. *ACS Appl. Nano Mater.* **2020**, *3* (7), 7088–7095.

(11) Xu, J.; Li, C.; Si, H.; Zhao, X.; Wang, L.; Jiang, S.; Wei, D.; Yu, J.; Xiu, X.; Zhang, C. 3D SERS Substrate Based on Au-Ag Bi-Metal Nanoparticles/MoS_2 Hybrid with Pyramid Structure. *Opt. Express, OE* **2018**, *26* (17), 21546–21557.

(12) Zhang, Y.; Wang, C.; Wang, J.; Chen, L.; Li, J.; Liu, Y.; Zhao, X.; Wang, Y.; Yang, J. Nanocap Array of Au:Ag Composite for Surface-Enhanced Raman Scattering. *Spectrochim. Acta, Part A* **2016**, *152*, 461–467.

(13) Wang, Z.; Wen, X.; Feng, Z.; Lin, L.; Liu, R.; Huang, P.; Chen, G.; Huang, F.; Zheng, Z. Highly Ordered Au-Ag Alloy Arrays with Tunable Morphologies for Surface Enhanced Raman Spectroscopy. *Chem. Eng. J.* **2018**, *345*, 389–394.

(14) Zhou, B.; Shen, J.; Li, P.; Ge, M.; Lin, D.; Li, Y. Y.; Lu, J.; Yang, L. Gold Nanoparticle-Decorated Silver Needle for Surface-Enhanced Raman Spectroscopy Screening of Residual Malachite Green in Aquaculture Products. *ACS Appl. Nano Mater.* **2019**, *2* (5), 2752–2757.

(15) Dou, X.; Zhao, L.; Li, X.; Qin, L.; Han, S.; Kang, S.-Z. Ag Nanoparticles Decorated Mesh-like MoS_2 Hierarchical Nanostructure Fabricated on Ti Foil: A Highly Sensitive SERS Substrate for Detection of Trace Malachite Green in Flowing Water. *Appl. Surf. Sci.* **2020**, *509*, 145331.

(16) Liu, Y.; Guan, H.; Lin, S.; Dong, H.; Hasi, W.; Dong, B. Plasmonic Nanosensor Based on Ag Nanocubes of High Purification by Extraction Filtration Strategy for SERS Determination of Malachite Green in Aquaculture Water. *Sens. Actuators, B* **2022**, *358*, 131515.

(17) Miao, Y.; Yang, K.; Zong, S.; Wang, Z.; Cui, Y. Au/Ag Bimetallic Nanocuboid Superlattices Coated with Ti_3C_2 Nanosheets for Surface-Enhanced Raman Spectroscopy Detection of Fish Drug Residues in Pond Water. *ACS Appl. Nano Mater.* **2021**, *4* (7), 6844–6851.

(18) Liu, Y.; Zhu, J.; Weng, G.; Li, J.; Zhao, J. Simultaneous determination of three food contaminants in shrimp paste by hollow porous pentagonal Au/Ag alloy nanotubes with excellent SERS activity. *Sens. Actuators, B* **2022**, *373*, 132766.

(19) Tzeng, Y.; Lin, B.Y. Silver SERS Adenine Sensors with a Very Low Detection Limit. *Biosensors* **2020**, *10* (5), 53.

(20) Pinheiro, P. C.; Fateixa, S.; Nogueira, H. I. S.; Trindade, T. SERS Study on Adenine Using a Ag/Poly(t-Butylacrylate) Nanocomposite. *Spectrochim. Acta, Part A* **2013**, *101*, 36–39.

(21) Zhou, N.; Meng, G.; Zhu, C.; Chen, B.; Zhou, Q.; Ke, Y.; Huo, D. A Silver-Grafted Sponge as an Effective Surface-Enhanced Raman Scattering Substrate. *Sens. Actuators, B* **2018**, *258*, 56–63.

(22) Madzharova, F.; Heiner, Z.; Gühlke, M.; Kneipp, J. Surface-Enhanced Hyper-Raman Spectra of Adenine, Guanine, Cytosine, Thymine, and Uracil. *J. Phys. Chem. C* **2016**, *120* (28), 15415–15423.

(23) Cao, J.; Liu, H.L.; Yang, J.M.; Li, Z.Q.; Yang, D.R.; Ji, L.N.; Wang, K.; Xia, X.H. SERS Detection of Nucleobases in Single Silver Plasmonic Nanopores. *ACS Sens.* **2020**, *5* (7), 2198–2204.

(24) Cho, K.H.; Choo, J.; Joo, S.W. Surface-Enhanced Raman Scattering and Density Functional Theory Calculation of Uracil on Gold and Silver Nanoparticle Surfaces. *Spectrochim. Acta, Part A* **2005**, *61* (6), 1141–1145.
